# Long-Term Evolution of *Burkholderia multivorans* during a Chronic Cystic Fibrosis Infection Reveals Shifting Forces of Selection

**DOI:** 10.1128/mSystems.00029-16

**Published:** 2016-05-24

**Authors:** Inês N. Silva, Pedro M. Santos, Mário R. Santos, James E. A. Zlosnik, David P. Speert, Sean W. Buskirk, Eric L. Bruger, Christopher M. Waters, Vaughn S. Cooper, Leonilde M. Moreira

**Affiliations:** a Institute for Bioengineering and Biosciences, Instituto Superior Técnico, Universidade de Lisboa, Lisbon, Portugal; b Centre of Molecular and Environmental Biology, Department of Biology, Universidade do Minho, Braga, Portugal; c Department of Pediatrics, Faculty of Medicine, Centre for Understanding and Preventing Infection in Children, University of British Columbia, Vancouver, Canada; d Department of Molecular, Cellular and Biomedical Sciences, University of New Hampshire, Durham, New Hampshire, USA; e Department of Microbiology and Molecular Genetics, Michigan State University, East Lansing, Michigan, USA; f Department of Microbiology and Molecular Genetics, University of Pittsburgh, Pittsburgh, Pennsylvania, USA; g Department of Bioengineering, Instituto Superior Técnico, Universidade de Lisboa, Lisbon, Portugal; UC Davis Genome Center

**Keywords:** *Burkholderia multivorans*, biofilm, c-di-GMP signaling, chronic infection, cystic fibrosis, evolution, lung function decline, respiratory infection, within-host adaptation

## Abstract

*Burkholderia multivorans* is an opportunistic pathogen capable of causing severe disease in patients with cystic fibrosis (CF). Patients may be chronically infected for years, during which the bacterial population evolves in response to unknown forces. Here we analyze the genomic and functional evolution of a *B. multivorans* infection that was sequentially sampled from a CF patient over 20 years. The population diversified into at least four primary, coexisting clades with distinct evolutionary dynamics. The average substitution rate was only 2.4 mutations/year, but notably, some lineages evolved more slowly, whereas one diversified more rapidly by mostly nonsynonymous mutations. Ten loci, mostly involved in gene expression regulation and lipid metabolism, acquired three or more independent mutations and define likely targets of selection. Further, a broad range of phenotypes changed in association with the evolved mutations; they included antimicrobial resistance, biofilm regulation, and the presentation of lipopolysaccharide O-antigen repeats, which was directly caused by evolved mutations. Additionally, early isolates acquired mutations in genes involved in cyclic di-GMP (c-di-GMP) metabolism that associated with increased c-di-GMP intracellular levels. Accordingly, these isolates showed lower motility and increased biofilm formation and adhesion to CFBE41o^−^ epithelial cells than the initial isolate, and each of these phenotypes is an important trait for bacterial persistence. The timing of the emergence of this clade of more adherent genotypes correlated with the period of greatest decline in the patient’s lung function. All together, our observations suggest that selection on *B. multivorans* populations during long-term colonization of CF patient lungs either directly or indirectly targets adherence, metabolism, and changes in the cell envelope related to adaptation to the biofilm lifestyle.

**IMPORTANCE** Bacteria may become genetically and phenotypically diverse during long-term colonization of cystic fibrosis (CF) patient lungs, yet our understanding of within-host evolutionary processes during these infections is lacking. Here we combined current genome sequencing technologies and detailed phenotypic profiling of the opportunistic pathogen *Burkholderia multivorans* using sequential isolates sampled from a CF patient over 20 years. The evolutionary history of these isolates highlighted bacterial genes and pathways that were likely subject to strong selection within the host and were associated with altered phenotypes, such as biofilm production, motility, and antimicrobial resistance. Importantly, multiple lineages coexisted for years or even decades within the infection, and the period of diversification within the dominant lineage was associated with deterioration of the patient’s lung function. Identifying traits under strong selection during chronic infection not only sheds new light onto *Burkholderia* evolution but also sets the stage for tailored therapeutics targeting the prevailing lineages associated with disease progression.

## Abstract (precis)

Bacteria may become genetically and phenotypically diverse during long-term colonization of cystic fibrosis (CF) patient lungs, yet our understanding of within-host evolutionary processes during these infections is lacking. Here we combined current genome sequencing technologies and detailed phenotypic profiling of the opportunistic pathogen *Burkholderia multivorans* using sequential isolates sampled from a CF patient over 20 years. The evolutionary history of these isolates highlighted bacterial genes and pathways that were likely subject to strong selection within the host and were associated with altered phenotypes, such as biofilm production, motility, and antimicrobial resistance. Importantly, multiple lineages coexisted for years or even decades within the infection, and the period of diversification within the dominant lineage was associated with deterioration of the patient’s lung function. Identifying traits under strong selection during chronic infection not only sheds new light onto *Burkholderia* evolution but also sets the stage for tailored therapeutics targeting the prevailing lineages associated with disease progression.

## INTRODUCTION

Bacteria have evolved many strategies to adapt within their hosts, such as changes in their surface antigens to evade the immune system, remodeling of the host epigenetic machinery, and disruption of host signaling (1–3). While some host-microbe interactions have been extensively studied, less is known about the evolutionary process of bacterial adaptation inside individual hosts. Bacterial infections of the respiratory airways of cystic fibrosis (CF) patients present a dynamic and highly relevant model for studying microbial adaptation. Feared pathogens include *Pseudomonas aeruginosa* and strains of the *Burkholderia cepacia* complex, as they are difficult to eradicate from the lungs of CF patients due, in part, to their intrinsic resistance to antimicrobial compounds (4). One of the first genome comparisons of longitudinally collected *P. aeruginosa* isolates recovered from infected CF patients led to the identification of mutations in genes important for host adaptation, such as *mexZ*, encoding a negative regulator of a multidrug efflux pump, and *lasR*, encoding the primary quorum-sensing regulator (5). More recently, by performing whole-genome sequencing of more than 400 *P. aeruginosa* isolates recovered from 34 CF patients, Marvig and colleagues identified 52 genes that convergently evolved, most of them involved in antibiotic resistance, motility, and biofilm formation (6).

Infection of CF patient airways by *B. cepacia* complex bacteria can be transient but in most cases leads to chronic colonization, causing a progressive deterioration of lung function. In a subset of patients, *B. cepacia* complex infection develops into the “cepacia syndrome,” characterized by fatal necrotizing pneumonia sometimes accompanied by bacteremia (7). Although many studies of putative virulence factors of the *B. cepacia* complex have been performed (reviewed in references [Bibr B8][Bibr B9][Bibr B11]), very few characterized the determinants of adaptation by these bacteria to CF patient lungs. A retrospective study of 112 *Burkholderia dolosa* isolates from 14 patients with CF who were infected in an epidemic outbreak over the course of 16 years (12) identified several bacterial genes that adaptively evolved by tracking recurrent patterns of mutations during infection of multiple individuals. These mutations affected important phenotypes, such as antibiotic resistance and expression of surface polysaccharides (12). In a subsequent study, sputum samples from five patients who had been infected with *B. dolosa* were analyzed for intraspecies diversity, which illustrated that several lineages may coexist for many years within a patient and that genes involved in outer membrane components, iron scavenging, and antibiotic resistance are under strong selection pressure (13).

The first comprehensive study covering more than 600 *B. cepacia* complex isolates from the environment and from chronic lung infection samples from 100 CF patients analyzed variation in colony mucoidy due to exopolysaccharide biosynthesis (14). Beyond demonstrating that all species of the *B. cepacia* complex can express the mucoid phenotype, it showed that sequential clonal isolates from 15 patients may undergo switches in this phenotype, the majority being mucoid-to-nonmucoid transitions in the species *Burkholderia multivorans* (9 patients) and *Burkholderia cenocepacia* (3 patients) (14). Further, patients colonized with nonmucoid isolates tended to exhibit more-rapid deterioration in lung function and poorer clinical outcome than those from whom mucoid isolates were recovered (15). *In vitro* studies have shown that mucoid-to-nonmucoid transitions in *B. cepacia* complex organisms may occur under stress conditions imposed by a long stationary phase, oxidative and osmotic stresses, and subinhibitory concentrations of antibiotics (16). As the molecular mechanisms underlying within-host genotypic and phenotypic variation are unknown, our aim was to identify genome-wide adaptations that could explain the long-term persistence of *B. cepacia* complex bacteria in CF patient lungs. Thus, we sequenced the genomes of 22 *Burkholderia multivorans* isolates recovered over 20 years of chronic colonization in a patient from whom isolates with a mucoid-to-nonmucoid transition had been obtained (14). This represents the first retrospective study of the genomic and phenotypic evolution of longitudinally collected *B. multivorans* specimens, which is notable because *B. multivorans* has recently been shown to be the predominant *Burkholderia* species infecting CF patients in Canada, the United States, New Zealand, and Europe (17–20). Our primary objective was to identify pathoadaptive mechanisms to this particular host environment. Here, we provide evidence that several *B. multivorans* clades coexisted at any given time but evolved at different rates. Mutations that defined new clades and others that evolved independently in multiple isolates affected a limited number of genes encoding regulatory proteins, lipid and amino acid metabolic enzymes, and proteins involved in envelope biogenesis. Overall, the evolution of this *B. multivorans* population was marked by periods of strong diversifying selection followed by periods of relative stasis. This dynamic suggests that monitoring these evolutionary and molecular patterns might be used to design responsive therapies designed to limit population diversity and disease progression.

## RESULTS

### Evolution of *B. multivorans* within the airway of a CF patient involves diversification yet a slow and steady accumulation of selected mutations.

The CF patient from whom *B. multivorans* isolates were recovered carried a complex genotype (*CFTR* ΔF508/*CFTR* I444S) and is one of the nine patients chronically colonized by *B. multivorans* in which a colony morphotype mucoid-to-nonmucoid transition was identified previously (14). This patient was hospitalized once due to CF-related respiratory illness but had been treated on multiple occasions with antibiotics, including cephalosporins, fluoroquinolones, β-lactams, azithromycin, and sulfamethoxazole-trimethoprim. During the course of this patient’s regular semiannual examinations, *B. multivorans* was first isolated in 1993 and recovered periodically until 2013, yielding a total of 22 isolates for analysis (see Table S1 in the supplemental material). In 1996, 2006, 2011, and 2013, two colonies of different morphologies were analyzed, but at all other times, only one isolate was evaluated. Although CF patient airways harbor numerous microorganisms that evade detection by culture-based methods, the information available from these methods showed that this patient was initially infected by *Staphylococcus aureus*, *Haemophilus influenzae*, and *Pseudomonas aeruginosa* (years, 1989 to 1993), followed by a period of coinfection of these microorganisms with *B. multivorans* (1993 to 1996). After that period, except for *Candida* and *Staphylococcus aureus* isolation, only *B. multivorans* was reported (Table S2).

10.1128/mSystems.00029-16.1 Table S1  Bacterial strains used in this study. Download Table S1, DOCX file, 0.04 MB. Copyright © 2016 Silva et al. 2016 Silva et al. This content is distributed under the terms of the Creative Commons Attribution 4.0 International license.

10.1128/mSystems.00029-16.2 Table S2  Different species isolated from our CF patient’s airways. Download Table S2, DOCX file, 0.04 MB. Copyright © 2016 Silva et al. 2016 Silva et al. This content is distributed under the terms of the Creative Commons Attribution 4.0 International license.

The analysis of the mucoid phenotype, assessed by quantification of the dry weight ethanol precipitate present in the culture supernatant, indicated that isolates BM5, BM12, and BM22 were the only ones unable to produce detectable amounts of exopolysaccharide (see Fig. S1 in the supplemental material). Despite some statistically nonsignificant variation in the total amounts, all the other isolates were considered highly mucoid.

10.1128/mSystems.00029-16.7 Figure S1  Isolates of *B. multivorans* obtained from the same persistently colonized CF patient over 20 years varied in exopolysaccharide (EPS) production. Exopolysaccharide production by all isolates (means ± standard deviations [SD] from at least three independent cell cultivations) was measured by determining the dry weight of the ethanol precipitate present in the culture supernatant. Download Figure S1, TIF file, 0.7 MB. Copyright © 2016 Silva et al. 2016 Silva et al. This content is distributed under the terms of the Creative Commons Attribution 4.0 International license.

**FIG 1:**
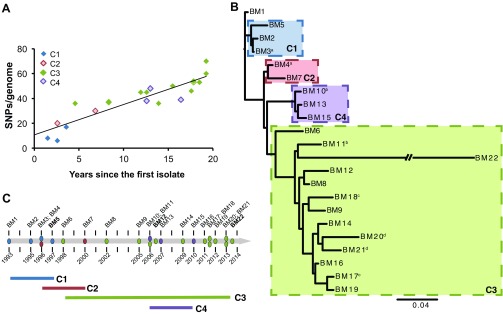
Genomic phylogeny reveals the long-term coexistence of diverse clades. Colors denote phylogenetic clades C1 to C4. (A) Number of SNPs distinguishing each isolate from the first (BM1) over time. The mutator isolate BM22 was excluded from this analysis. A linear fit with a slope of 2.4 mutations per year is shown. (B) Maximum *a posteriori* phylogenetic tree of the 22 isolates modeling both SNPs and indels as independent mutational processes. Shared superscripts in the strain labels indicate that isolates were obtained on the same date. (C) The temporal distribution of isolate samplings enables the inference that clades coexisted. Isolate labels in bold font are nonmucoid (see Fig. S1 in the supplemental material).

Aiming to understand the evolutionary adaptation of *B. multivorans* in the CF host environment and to identify genetic mutations possibly leading to phenotypic variation, we sequenced and analyzed the genomes of the 22 isolates. Single-nucleotide polymorphisms (SNPs) and indels were identified by mapping sequence reads for each isolate against the genome sequence of the first isolate (BM1). A total of 399 SNPs and 88 indels were detected over 20 years of chronic colonization (see Table S3 in the supplemental material). The last isolate (BM22) alone accounted for 196 new mutations, likely due to a frameshift mutation in the mismatch repair gene *mutL* which conferred a hypermutator phenotype. The estimated mutation rate of all isolates was 4.0 SNPs/year (*r* = 0.6), but if the last isolate is excluded, this rate drops to 2.4 (*r* = 0.9) (Fig. 1A), which is within the range observed for other long-term infections with *P. aeruginosa* and *B. dolosa* in CF patients (5, 12). We used both SNPs and indels as independent mutational processes (21) to determine the evolutionary relationship among the 22 isolates (Fig. 1B). The Bayesian maximum *a posteriori* phylogenetic tree revealed the evolution of an early clade, C1, from the first isolate, followed shortly by a lineage that quickly diversified into clades C2, C3, and C4. Note that this analysis produces an unrooted tree that nevertheless points to the node between BM1 and C1 as the common ancestor, and it reveals that indels resolve the phylogeny more completely than an SNP-based tree, which places the roots of C2 to C4 in an unresolved polytomy. The distribution of these clades over time (Fig. 1C) suggests that at least 2 to 3 distinct lineages coexisted at any given time, although the last 7 isolates were all from clade C3. Moreover, isolates BM3/BM4 and BM10/BM11, each a pair that was recovered simultaneously, clustered in different clades in the phylogenetic tree, consistent with the coexistence of different lineages within the same infected CF patient. These results provide further compelling evidence that sampling single clones from CF patient airways over time significantly undersamples the diversity within the prevailing infection.

10.1128/mSystems.00029-16.3 Table S3  Mutations found in all isolates. Download Table S3, XLSX file, 0.1 MB. Copyright © 2016 Silva et al. 2016 Silva et al. This content is distributed under the terms of the Creative Commons Attribution 4.0 International license.

### Early diversification results from strong selection on genes affecting regulatory functions, metabolism, and envelope biogenesis.

**FIG 2:**
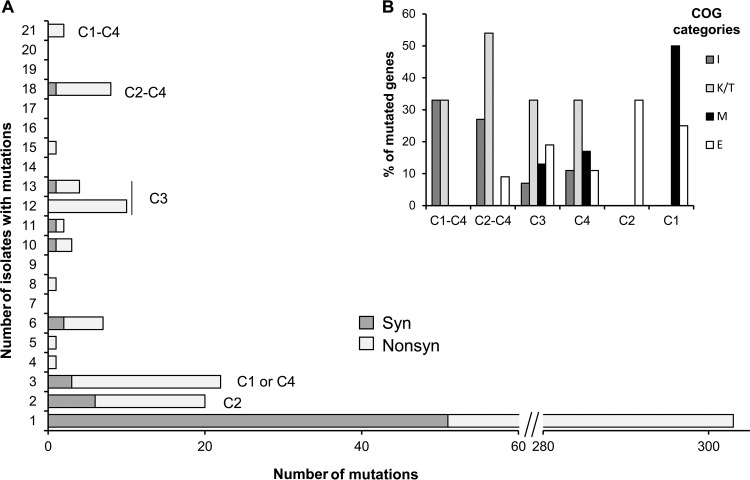
Most mutations were nonsynonymous, especially at the nodes of new clades, with affected genes encoding regulatory functions, lipid metabolism, and envelope biogenesis. (A) Numbers of synonymous and nonsynonymous mutations shared by each of the isolates and associated with nodes in the phylogeny (Fig. 1B); (B) distribution of the synonymous and nonsynonymous mutations at each node by clusters of the following orthologous groups: lipid transport and metabolism (COG I), transcription and signal transduction (COGs K and T), cell wall/membrane/envelope biogenesis (COG M), and amino acid transport and metabolism (COG E).

We identified mutations associated with each node that defined the four clades in the phylogenetic tree (Fig. 1B and 2A, and see Table S4 in the supplemental material). Most mutations, especially those defining clades and shared among multiple isolates, are nonsynonymous (Fig. 2A) (Fisher exact test, one tailed, *P* = 0.0155). Of the 64 mutations associated with lineage splitting, 41 were either nonsynonymous or indels, 5 were synonymous, and 18 were intergenic, most of which were proximal to coding sequences (CDSs) and hence near promoters. This enrichment of nonsynonymous coding mutations (41/46) is significantly greater than the expectation of 27.8% synonymous mutations under neutral evolution given the codon usage and percent G+C content of this species (22). This suggests that the infection involved periods of diversification dominated by positive selection followed by intervals of relatively neutral evolution.

10.1128/mSystems.00029-16.4 Table S4  (First tab) Mutations defining clades (C1 and C2, C3 and C4) are clustered by the nodes that they define. Their classification according to clusters of orthologous groups (COGs) is also included. (Second tab) List of polymorphic genes. Download Table S4, XLSX file, 0.05 MB. Copyright © 2016 Silva et al. 2016 Silva et al. This content is distributed under the terms of the Creative Commons Attribution 4.0 International license.

The nodes at the base of the four clades were enriched in functional categories that shifted over time. The first mutations shared by all clades, or all except C1, were enriched in mutations (SNPs and indels) in the categories of transcription/signal transduction (clusters of orthologous groups [COGs] K and T) and lipid metabolism (COG I) (Fig. 2B; Table S4 in the supplemental material). Mutations producing clades C3 and C4 were also enriched in transcription/signal transduction mutations (COGs K and T) but also in amino acid transport and metabolism (COG E) and cell wall/membrane/envelope biogenesis (COG M) mutations. The mutations at the roots of clades C1 and C2 affected genes from COGs E and M, and COG E, respectively (Fig. 2B; Table S4). This analysis reveals that new lineages evolved mainly by mutations in genes with regulatory/signaling roles (31.3%) and in genes whose proteins are involved in lipid, amino acid, and carbohydrate metabolism (32.8%), possibly having an impact on envelope biogenesis.

The most obvious candidates for genes under selection during this chronic *B. multivorans* colonization are mutations that became fixed over time. Only three mutations were found in all isolates from clades C1 to C4, including a nonsynonymous mutation in the gene encoding a FabD homolog (a malonyl coenzyme A [CoA]:acyl carrier protein [ACP] transacylase) involved in fatty acid biosynthesis, an insertion of 76 amino acids into cell division protein FtsK, and a point mutation 181 bp upstream of the coding region of a DNA-binding regulatory protein (Table 1). Twelve mutations were found in all isolates from clades C2, C3, and C4. These include genes encoding the diguanylate cyclase/phosphodiesterase RpfR (also known as YciR), which is also the receptor of the *Burkholderia* diffusible signal factor (BDSF), the subunit beta of acetyl-CoA carboxylase, which is involved in fatty acid biosynthesis, four proteins involved in transcription/signal transduction, and two transporters, including a multidrug resistance efflux pump membrane protein homolog to EmrA. Three other mutations were in intergenic putative-promoter regions, two of them upstream of the coding sequence of protein PlsX, involved in the biosynthesis of phospholipids, and another one upstream of the coding sequence of a OmpR-like response regulator (Table 1). A last mutation common to all isolates from clades C2 and C3 was a frameshift within gene *wbiI* (BMD20_17700), encoding a nucleoside diphosphate sugar epimerase involved in the synthesis of the O-antigen repeats of the lipopolysaccharide (LPS). Thus, two haplotypes, one derived from the other, rose to high frequency within this infection in its first 3 years.

**TABLE 1:** List of mutations that fixed in the infecting population and their association with phylogenetic clades C1 to C4, as shown in Fig. 1^a^

Gene locus	Annotation	Mutation category	Type of mutation	Effect in protein	Presence of mutation in clade:			
					C1	C2	C3	C4
BMD20_16520	ACP *S*-malonyltransferase FabD	CDS	SNP, nonsyn	Y89H	+	+	+	+
BMD20_24750	Cell division protein FtsK	CDS	Indel, 76-aa insertion		+	+	+	+
BMD20_24880	DNA-binding regulatory protein, YebC/PmpR family	INT (181-bp upstream CDS)	SNP		+	+	+	+
BMD20_02495	Diguanylate cyclase/phosphodiesterase RpfR	CDS	SNP, nonsyn	E185D	−	+	+	+
BMD20_04285	Acetyl-CoA carboxylase, carboxyl transferase subunit beta AccD	CDS	SNP, nonsyn	T240M	−	+	+	+
BMD20_05445	Transcriptional regulator/2-aminoadipate aminotransferase	CDS	SNP, syn	A362A	−	+	+	+
BMD20_06065	Two-component heavy-metal response transcriptional regulator	CDS	SNP, nonsyn	E229D	−	+	+	+
BMD20_08755	LysR transcriptional regulator	CDS	SNP, nonsyn	A24E	−	+	+	+
BMD20_10060	DNA-binding protein	CDS	SNP, nonsyn	I228M	−	+	+	+
BMD20_11660	OmpR-like response regulator	INT (16-bp upstream CDS)	SNP		−	+	+	+
BMD20_14360	ABC transporter permease	CDS	SNP, nonsyn	P12T	−	+	+	+
BMD20_14695	Multidrug resistance efflux pump membrane protein EmrA	CDS	SNP, nonsyn	Q97H	−	+	+	+
BMD20_16530	Glycerol-3-phosphate acyltransferase PlsX	INT (9-bp upstream CDS)	SNP		−	+	+	+
BMD20_16530	Glycerol-3-phosphate acyltransferase PlsX	INT (10-bp upstream CDS)	SNP		−	+	+	+
BMD20_17700	Nucleoside-diphosphate sugar epimerase WbiI	CDS	Indel, frameshift		−	+	+	−

a CDS, coding sequence; INT, intergenic region; aa, amino acid; nonsyn, nonsynonymous; syn, synonymous.

**FIG 3:**
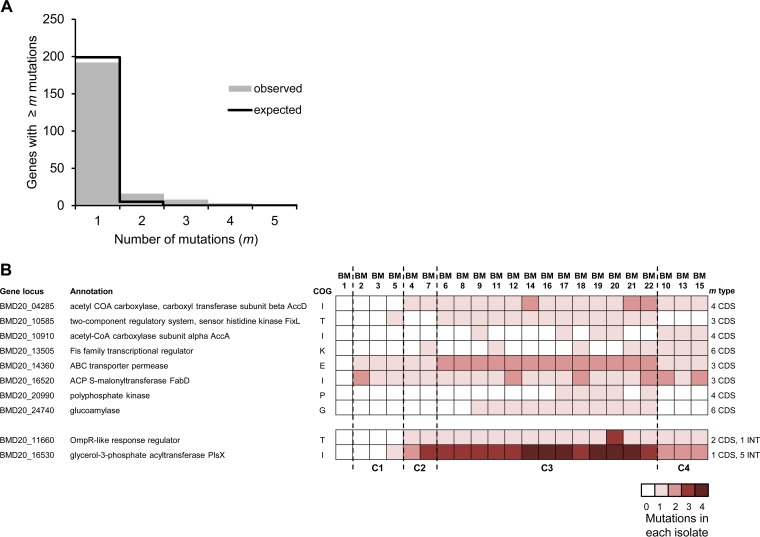
Strong parallelism in mutated genes defines adaptations to the environment of CF patient lungs. (A) Observed and expected numbers of genes with at least *m* mutations given the number of total mutations found. Genes with multiple mutations are in significant excess (*P* < 0.005). (B) Heatmap of mutation frequencies in the 10 loci that acquired ≥3 mutations, including point mutations upstream of the coding sequence that might be relevant for gene expression. Isolates are clustered by clade. (Right) Total numbers of mutations acquired per gene (*m*) and the type of mutation (coding sequence [CDS] or intergenic [INT]).

However, the 41 genes in which we detected multiple independent mutations may provide even stronger and more informative evidence of selection, given that some mutations at nodes may have risen to high frequency by linkage to another selected mutation and not by any directly selected phenotype. Of the 218 independent mutations found in all isolates except the last hypermutator isolate, we expected none to occur in the same gene three or more times by chance (*P* < 0.005) among the 5,809 genes in this *B. multivorans* genome (Fig. 3A). Notably, eight genes were found to have three or more different mutations, five genes had four or more mutations, and two genes had five or more mutations. Eight of these polymorphic genes with three or more mutations within the coding sequence and two other genes with mutations upstream of the CDS are detailed in Fig. 3B (see also Table S4 in the supplemental material). Three of these polymorphic genes encode proteins involved in regulating transcription and signal transduction that may influence pathogenesis. One gene with six mutations (BMD20_13505, with 5 nonsynonymous mutations and 1 frameshift mutation) that evolved independently in clades C2, C3, and C4 encodes a Fis family transcriptional regulator conserved across the *Burkholderia* genus, but the genes that it regulates are currently unknown. Two genes with three mutations were the *fixL* homolog BMD20_10585 and the *ompR*-like response regulator of a two-component signal transduction system (BMD20_11660). BMD20_10585 is a homolog of BDAG_01161 from *B. dolosa*, the *fixL* homolog that accumulated the highest number of mutations in the 112 clinical isolates analyzed by Lieberman and colleagues (12). Homologs of this gene have been implicated in oxygen sensing in other microorganisms (23), and due to the low-oxygen tension of CF patient lungs, it is possible that alterations in this *Burkholderia* regulator are involved in adaptation to microaerophilic conditions. Another gene, BMD20_11660, encodes a response regulator homologous to the osmolarity response regulator OmpR and is located upstream of a region encoding a sensor signal histidine kinase. Multiple mutations in the ortholog of this locus in *B. cenocepacia* populations were associated with adaptation during long-term experimental evolution in a biofilm system (24). Although the genes regulated by this two-component signal transduction system are unknown, we demonstrate that the point mutation 16 nucleotides upstream of the start codon increased the transcription of the BMD20_11660 gene in two isolates tested from clade C3 and one from clade C4 relative to that of isolate BM1 or BM2, which lacked any mutation (Table S5). To summarize, mutations in genes associated with regulation of the cellular response to reduced oxygen levels and perhaps also to osmolarity were under selection in multiple independent isolates.

10.1128/mSystems.00029-16.5 Table S5  Quantitative real-time RT-PCR analysis of the BMD20_11660 gene of *B. multivorans* isolates relative to the first isolate, BM1. Download Table S5, DOCX file, 0.03 MB. Copyright © 2016 Silva et al. 2016 Silva et al. This content is distributed under the terms of the Creative Commons Attribution 4.0 International license.

The second group of genes that acquired 3 to 6 different mutations (encoding acetyl-CoA carboxylase subunits alpha and beta, the FabD homolog, and the glycerol-3-phosphate acyltransferase PlsX) are all involved in the metabolism of fatty acids/phospholipids. Particularly notable are the six mutations that accumulated in the *plsX* gene (1 nonsynonymous mutation and 5 point mutations in the untranslated region upstream of the start codon). Three other genes that acquired multiple mutations encode an ABC transporter permease (3 mutations), a glucoamylase (4 nonsynonymous point mutations and 2 frameshift mutations, all in clade C3 isolates) that was also mutated in *B. dolosa* isolates (12), and polyphosphate kinase (all mutations are in clade C3 isolates), an enzyme important for virulence and response to stress in many bacterial pathogens (25). In summary, the coexisting subpopulations within this infection each faced similar selective pressures favoring independent mutations in several regulatory and metabolic genes.

### The first-evolved phenotypes included reduced growth rates and altered O-antigen repeats.

Given strong genetic evidence of positive selection during the infection, we sought to determine the associated mutant phenotypes that could have been selected. First, the *in vitro* doubling times and biomass yields of the isolates were estimated in synthetic cystic fibrosis medium. Compared to the doubling times of 6 h for isolate BM1 and of 6.2 to 6.3 h for clade C4 isolates, all isolates from clades C1 (*P* < 0.01), C2, and C3 (*P* < 0.05) needed between 7 and 15 h to duplicate (see Fig. S2A in the supplemental material). These differences are even more pronounced for biomass, in which all isolates after BM1 exhibited reduced output (*P* < 0.001) (Fig. S2B). Only four mutations distinguish isolate BM1 from all subsequent isolates, implying that at least one is responsible for this slow-growth phenotype according to the logic of parsimony. It remains possible that multiple causes of slow growth may have evolved, however. One such mutation likely to influence growth is a 76-amino-acid insertion at position 220 of protein FtsK, a septum-located DNA translocase that coordinates the late stages of cytokinesis and chromosome segregation during cell division. The other mutations altered a noncoding region upstream of an uncharacterized DNA-binding protein similar to YebC/PmpR, a malonyl CoA:acyl carrier protein (ACP) transacylase, and an ABC transporter permease possibly transporting amino acids. Multiple independent mutations involved this permease gene, which is evidence of strong selection for a putative transport function.

10.1128/mSystems.00029-16.8 Figure S2  Changes in the growth rate are among the first-evolved phenotypes. Doubling times (A) and biomasses (OD_640nm_) (B) of the isolates grown in SCFM at 37°C. Data are the means of results from three independent experiments. The doubling times of all clades (except C4) and final biomasses differed significantly from those of BM1 (*, *P* < 0.05; **, *P* < 0.01; ***, *P* < 0.001) by Tukey’s HSD multiple-comparison test for unequal group sample sizes. Download Figure S2, TIF file, 0.6 MB. Copyright © 2016 Silva et al. 2016 Silva et al. This content is distributed under the terms of the Creative Commons Attribution 4.0 International license.

**FIG 4:**
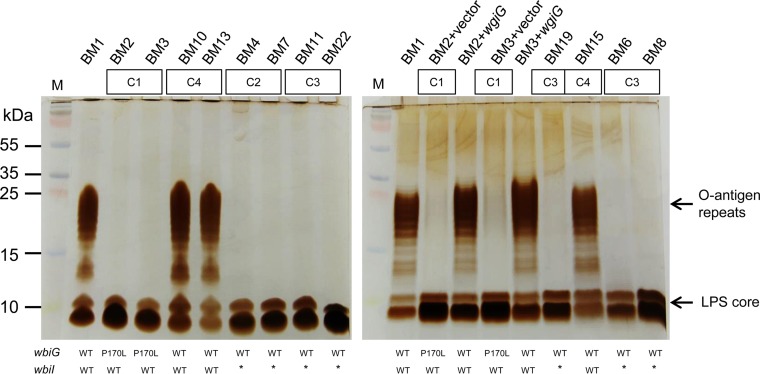
Evolved changes in the LPS pattern and the causative mutation. (Left) Electrophoretic profiles of LPSs extracted from *B. multivorans* isolates representative of all clades and *wbi* alleles, in which the *wgiG* mutation resulting in P169L and a frameshift mutation in *wbiI* are associated with the loss of the O antigen. (Right) BM2 and BM3 were complemented with the empty vector (pBBR1MCS) or a vector carrying a functional *wgiG* copy from isolate BM1, which restores the O antigen. Lanes M, protein markers. WT, wild-type allele; *, *wbiI* frameshift mutation.

The finding of three different mutations accumulating in a locus homologous to the *wbi* gene cluster led us to predict that biosynthesis of the LPS O antigen had been altered (26). LPS plays an important role in *Burkholderia* virulence, and O-antigen repeats are known to interfere with adhesion to abiotic surfaces and bronchial epithelial cells (27, 28). One mutation in the *wbiG* gene (BMD20_17710), a nucleotide sugar epimerase/dehydratase, was found in all isolates from clade C1 (BM2, BM3, and BM5). This nonsynonymous mutation altered the conserved region ^168^PPLV^173^ to ^168^PLLV^173^. We assessed whether this mutation altered the LPS O-antigen repeats in the bacterial outer membrane and found that the two tested isolates from clade C1 do not produce the O-antigen repeats of LPS but that isolate BM1 presents a smooth LPS (Fig. 4). Complementation of BM2 and BM3 isolates with the BM1 *wbiG* allele expressed from pBBR1MCS under the native promoter restored the O-antigen repeats in those isolates (Fig. 4). Another mutated gene was *wbiI* (BMD20_17700), encoding another nucleotide sugar epimerase/dehydratase. Deletion of one nucleotide in *wbiI* occurred in all isolates from clades C2 and C3 and led to a frameshift mutation. As predicted, representative isolates from clades C2 and C3 also lacked the O-antigen portion of LPS (Fig. 4). However, the three isolates from clade C4 had no *wbi* mutations, potentially by reversion of the *wbiI* frameshift mutation, and displayed the same LPS O-antigen pattern as isolate BM1 (Fig. 4). Further, isolate BM22 has a nonsynonymous mutation in *wbiF* (BMD20_17715), encoding a glycosyltransferase, in addition to the *wbiI* mutation. We conclude that clones with mutations in the *wbi* locus were favored during selection and produced altered LPS O antigen.

### Evolutionarily successful lineages originated from mutations affecting antibiotic resistance and cyclic-diguanylate signaling.

**FIG 5:**
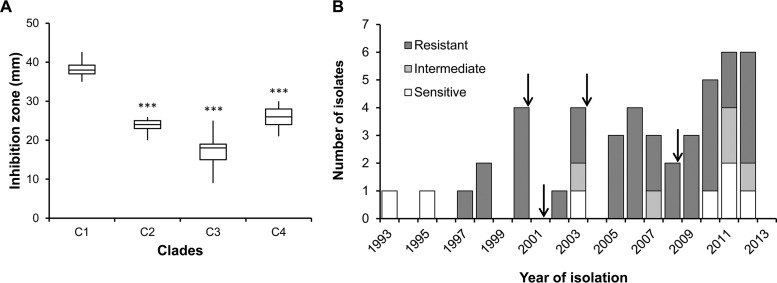
The evolution of antibiotic resistance associates with a mutation in an efflux pump. (A) Susceptibilities to piperacillin plus tazobactam determined at 37°C after 24 h of incubation by measuring the diameter of cell growth inhibition. Isolates from clades C2, C3, and C4 have the *emrA* mutation. Clade C1 isolates (including also isolate BM1) lacking the mutation differed significantly from those with the mutation (***, *P* < 0.001) by Tukey’s HSD multiple-comparison test for unequal group sample sizes. (B) Temporal variations in levels of resistance to piperacillin plus tazobactam of all *B. multivorans* colonies recovered from the CF patient under study. Arrows indicate the date that the patient was subjected to an antibiotic therapy.

The phylogeny (Fig. 1B) shows that most of the evolved diversity and all successful lineages trace to the root of clades C2, C3, and C4, which dates to the first 2.5 years of the *B. multivorans* infection. There are 12 mutations at this node (7 nonsynonymous, 1 synonymous, and 4 noncoding), which makes identifying the driver(s) of this diversification challenging. One potential and obvious target of selection is increased antimicrobial resistance. Among the 12 mutations at this node, one (Q97H) occurred in BMD20_14695, a homolog of the *emrA*/*hlyD* gene encoding the periplasmic adapter protein (also known as the membrane fusion protein) of a bacterial tripartite efflux pump putatively involved in the extrusion of toxic compounds (29). To test this hypothesized function, the different isolates were assayed for resistance to piperacillin plus tazobactam, a combined antibiotic therapy frequently prescribed to individuals with cystic fibrosis. Although piperacillin and tazobactam are a β-lactam and a β-lactamase inhibitor, respectively, *Burkholderia* resistance against these antibiotics may involve efflux pumps rather than β-lactamases, as has been suggested for *P. aeruginosa* (30). Compared to that of BM1 and the early clade C1 isolates, resistance among the 18 isolates harboring the mutated allele of the *emrA* gene was increased (*P* < 0.001) (Fig. 5A). The Q97H mutation maps to the beginning of the α-helical hairpin domain, which in other EmrA homologs has a coiled-coil arrangement for interaction with TolC-like outer membrane proteins to form sealed periplasmic channels (31). Alignment of the amino acid sequence of EmrA with the Q-to-H mutation at position 97 (EmrA_Q97H_) to sequences of other *Burkholderia* homologs showed the replacement of Q97 by the positively charged amino acids R or K, but never H. It remains to be determined whether this mutation is the basis for the increased resistance to piperacillin plus tazobactam of isolates from clades C2, C3, and C4. Consistently with this data, the antimicrobial susceptibilities to piperacillin plus tazobactam of the *B. multivorans* isolates under study and of the discarded ones carried out by the clinical laboratory showed that the first isolates were susceptible, followed by a period during which the majority were resistant (Fig. 5B). The arrows in Fig. 5B indicate the periods of antimicrobial therapy administration to this patient. Interestingly, following antibiotic therapy, only resistant bacteria were isolated, but in the subsequent 1 to 3 years of no-antibiotic pressure, some susceptible isolates were again recovered. Similar trends were observed with other antibiotics (see Fig. S3 in the supplemental material).

10.1128/mSystems.00029-16.9 Figure S3  Antibiotic resistance profiles of the *B. multivorans* isolates recovered from the CF patient under study between 1993 and 2013. Isolates were tested for the indicated antibiotics and classified by the clinical laboratory as resistant, intermediate, or sensitive. Arrows indicate the dates that the patient was started an antibiotic therapy as indicated in the chart review, namely, September 2000, June 2001, November 2003, and May 2008. Download Figure S3, TIF file, 1.4 MB. Copyright © 2016 Silva et al. 2016 Silva et al. This content is distributed under the terms of the Creative Commons Attribution 4.0 International license.

**FIG 6:**
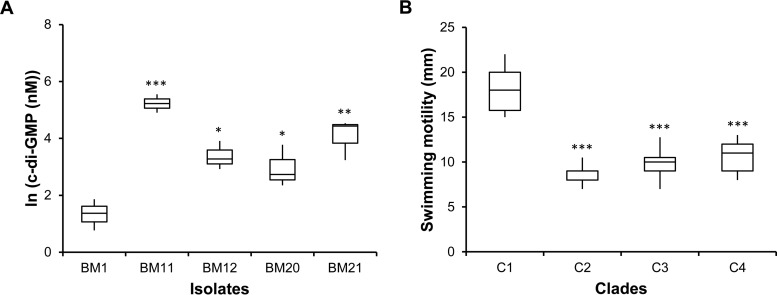
Later isolates from the dominant clade, C3, exhibit higher c-di-GMP levels and less motility than earlier isolates. (A) Intracellular c-di-GMP concentrations in BM1 and clade C3 (BM11, BM12, BM20, and BM21) isolates following 5 h of growth, as measured by LC-MS/MS. Significantly greater c-di-GMP levels were detected in evolved isolates than in the first isolate, BM1 (*, *P* < 0.05; **, *P* < 0.01; ***, *P* < 0.001), by Tukey’s HSD multiple-comparison test. (B) Swimming motility (assayed in 0.3% of agar plates incubated at 37°C for 24 h) was significantly less for isolates from clades C2 to C4 than for isolates from C1 (which includes isolate BM1) (***, *P* < 0.001 by Tukey’s HSD multiple-comparison test).

Another mutation at the node preceding the diversification of clades C2, C3, and C4 and thus shared by all derived isolates arose in gene BMD20_02495, the *rpfR* homolog that encodes the cis-2-dodecenoic acid (BDSF) receptor (Table S4). This protein is involved in cyclic di-GMP (c-di-GMP) metabolism mainly as a diguanylate phosphodiesterase, which has been shown to promote motility and reduced biofilm formation in *Burkholderia cenocepacia* (24, 32, 33). This mutation is the only 1 of the 12 at this node with a clear connection to c-di-GMP metabolism. The E185D amino acid substitution occurred at a strictly conserved glutamate residue (see Fig. S4 in the supplemental material) that is part of the Per-Arnt-Sim (PAS) or BDSF-sensing domain. As BDSF molecules have been shown to bind the PAS domain of *B. cenocepacia* RpfR and stimulate its phosphodiesterase activity (34, 35), alteration of this domain can alter affinity to BDSF signaling and consequently disrupt the phosphodiesterase activity of RfpR. We therefore quantified levels of c-di-GMP in cells grown for 5 h in salt-mannitol (SM) medium (16). As predicted, all tested isolates with the RpfR_E175D_ mutation exhibited increased c-di-GMP levels (*P* < 0.05 for BM12 and BM20; *P* < 0.01 for BM21; *P* < 0.001 for BM11) (Fig. 6A). However, two tested isolates (BM12 and BM21) acquired subsequent mutations in genes with putative diguanylate cyclase (GGDEF) or phosphodiesterase (EAL) domains, which may also have altered c-di-GMP levels, but this compensatory phenotype has not been tested. Because deleting RpfR reduces *B. cenocepacia* motility (35), we also measured this trait for these isolates. Figure 6B confirmed the decrease in swimming motility of the isolates harboring the RpfR_E175D_ mutation (*P* < 0.001), suggesting that increased levels of c-di-GMP reduce swimming motility.

10.1128/mSystems.00029-16.10 Figure S4  Multiple-sequence alignment of RpfR from *B. multivorans* BM11. Only amino acids at position 145 to 194 from BMD20_02495 are shown. The arrow indicates the glutamate residue replaced in BM11 by aspartate. Row 1, *Pandoraea* sp. strain B-6; row 2, *Bordetella* sp. strain FB-8; row 3, *Burkholderia contaminans* LMG 23361; row 4, *Burkholderia contaminans* FFH2055; row 5, *Burkholderia lata*; row 6, *Burkholderia pyrrocinia*; row 7, *Burkholderia cenocepacia* H111; row 8, *Burkholderia cepacia*; row 9, *Burkholderia multivorans* D2095 (BM11); row 10, *Burkholderia multivorans* ATCC 17616; row 11, *Burkholderia dolosa*; row 12, *Burkholderia ambifaria* IOP40-10; row 13, *Burkholderia vietnamiensis* G4; row 14, *Burkholderia ubonensis*; row 15, *Pandoraea thiooxydans*; row 16, *Burkholderia caribensis* MBA4; row 17, *Burkholderia terrae*; row 18, *Burkholderia phymatum* STM815; row 19, *Burkholderia caledonica*; row 20, *Burkholderia bryophila*; row 21, *Burkholderia fungorum*; row 22, *Burkholderia kururiensis*; row 23, *Achromobacter arsenitoxydans* SY8; row 24, *Serratia liquefaciens*; row 25, *Enterobacter cloacae*; row 26, *Yersinia mollaretii*. Download Figure S4, TIF file, 1.7 MB. Copyright © 2016 Silva et al. 2016 Silva et al. This content is distributed under the terms of the Creative Commons Attribution 4.0 International license.

### Increased biofilm formation and adhesion to epithelial cells play a role in bacterial adaptation within CF patient lungs.

**FIG 7:**
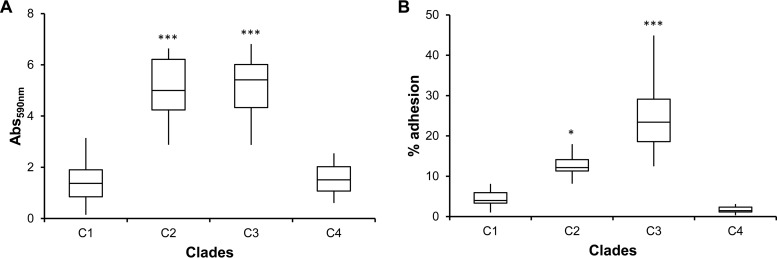
Biofilm formation and adhesion to cystic fibrosis bronchial epithelial cells increase during long-term colonization of CF patient lungs. Biofilm formation (A) and adhesion to CFBE41o^−^ cystic fibrosis lung epithelial cells (B) increase for clade C2 and C3 isolates (*, *P* < 0.05; ***, *P* < 0.001 by Tukey’s HSD multiple-comparison test). Clade C1 also includes isolate BM1.

Since c-di-GMP is known to regulate several virulence determinants, including biofilm formation (36–41), we quantified biofilm production by all isolates (Fig. 7A). Isolates from clades C2 and C3 produce more biofilm than the first isolate as well as isolates from clades C1 and C4 (*P* < 0.001). Many factors beyond c-di-GMP levels, including adhesion mechanisms, the biosynthesis of various polymers, quorum-sensing signaling, and LPS (38), contribute to biofilm formation. The higher levels of c-di-GMP in clade C3 isolates and possibly also in C2 and C4 isolates (all harboring RpfR_E185D_ and mutations in lipid biosynthesis metabolic enzymes) correlated with the observed increase in biofilm biomass, except in isolates BM10, BM13, and BM15 from clade C4. Nevertheless, clade C4 isolates display a smooth LPS in which the O-antigen repeats can possibly mask surface structures affecting the adhesion of bacteria to surfaces, as was reported for *Burkholderia pseudomallei* (42).

In agreement with the biofilm assays on polystyrene, isolates from clades C2 and C3 also adhered better to cystic fibrosis patient bronchial epithelial CFBE41o^−^ cells (*P* < 0.001 for C3 and *P* < 0.05 for C2) than did isolate BM1 and clade C1 isolates (Fig. 7B). Again, isolates from clade C4 were the least adherent, potentially owing to the presence of the LPS O-antigen repeats. In *B. cenocepacia*, the lipopolysaccharide O antigen prevents adhesion to epithelial cells (27). In summary, variation in biofilm production and adherence to epithelial cells were likely traits selected to increase during this chronic infection, but notably, variation in adherence was observed throughout the infection.

### The highest rate of lung function deterioration parallels genotypic and phenotypic diversification of the isolates and alteration of the lung microbiota.

**FIG 8:**
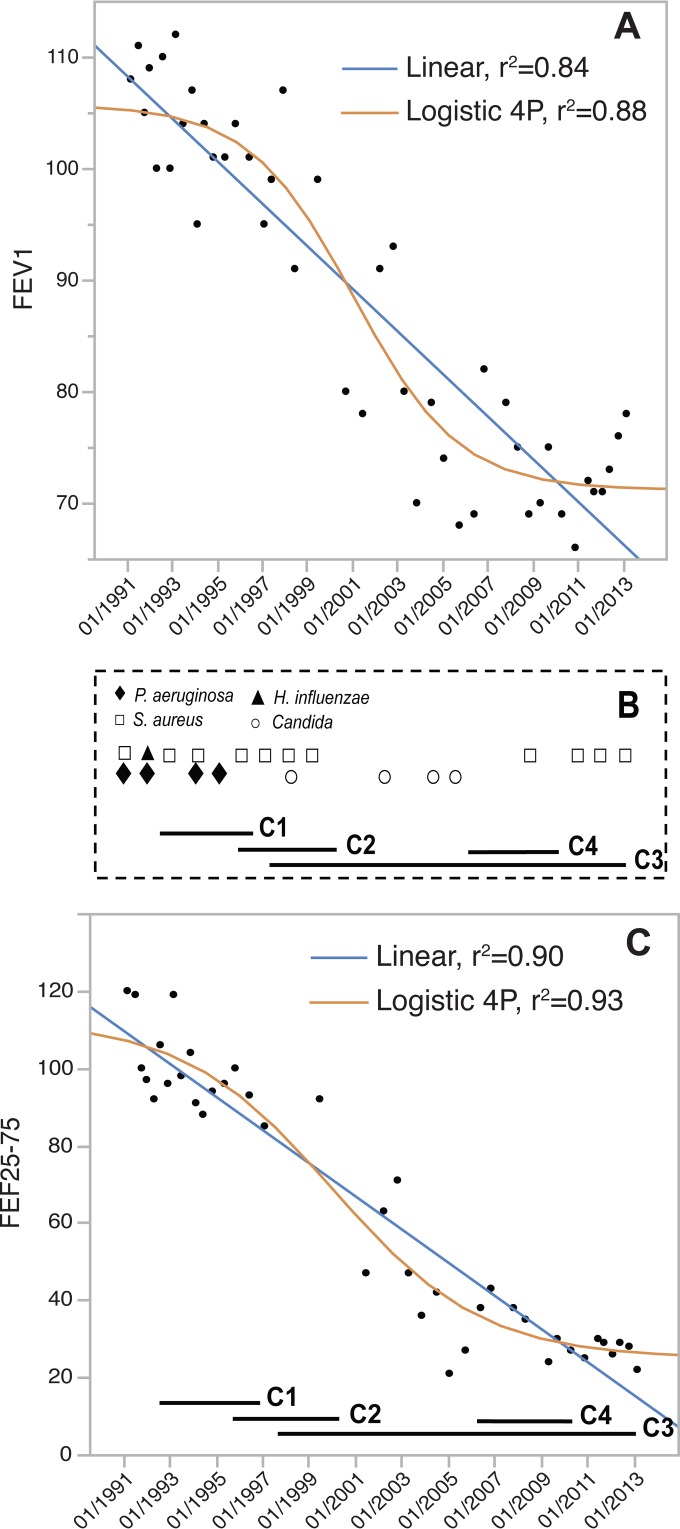
Lung function decline correlates with the evolution of clade C3 and mutations influencing LPS biosynthesis, motility, adhesion to surfaces, and cyclic diguanylate signaling. Forced expiratory volume in 1 s (FEV_1_) (A) and forced expiratory flow over the middle half of the forced vital capacity (FEF25−75%) (C) as predicted percentages. Patient respirometry data and clinical microbiological reports from sputa were collected during a previous retrospective study (15) and were available 2 years prior to the detection of *B. multivorans* and in the subsequent 20 years of infection. Both linear and 4-parameter (4P) logistic functions (shown), as well as quadratic, cubic, and logistic 3-parameter and 5-parameter models (not shown), were fit to the data and evaluated for the greatest quality using the Akaike information criterion (AIC). The upward trend in FEV_1_ in the last 2 years slightly favored a cubic fit over other models (FEV_1_, linear AIC = 296.5, quadratic AIC = 292.4, logistic 5 parameters = 288.5, logistic 4 parameters = 287.2, cubic = 285.1), whereas the logistic 4-parameter model provided the best fit for FEF25−75% (linear = 307.5, quadratic = 303.0, logistic 5 parameters = 300.5, cubic = 299.4, logistic 4 parameters = 298.1 [lower is better]). This supports a period of more rapid decline consistent with the rise of clade C3. (B) Temporal distribution of microorganisms (see Table S2 in the supplemental material) coinfecting the lungs of the CF patient under study, as well as the phylogenetic structure of the detected *B. multivorans* isolates.

The vital lung capacity of this patient, as determined by forced expiratory volume in 1 s (FEV_1_) (percentage predicted) showed a 1.9% decline per year, indicative of a mild infection. Nevertheless, the plot of FEV_1_ over time apparently followed three periods with different rates of decline (Fig. 8A). To test this interpretation, we fit multiple continuous models to the temporal FEV_1_ measures for comparison to a linear model and evaluated for greatest quality using the Akaike information criterion (AIC) and JMP v.11.0. We found that a cubic function producing a sigmoidal curve, followed closely by a four-parameter logistic function, provided a better fit to the data than a linear function or any other models (3- or 5-parameter logistic functions or quadratic functions) (see the legend of Fig. 8A). This supports a period of more rapid decline consistent with the rise of clade C3 isolates. In the first interval from 1989 to 1997, FEV_1_ declined 1.2% per year, in which isolates from clade C1 prevailed in coinfections with *P. aeruginosa* and *Staphylococcus aureus* (Fig. 8B). Between 1998 and 2006 the decline was 3.2% per year and corresponds to the rise of clade C3 isolates. The cooccurring pathogens during this period also differed, with frequent detection of *Candida*. After 2006, the decline was 0.7% per year, corresponding to the sampling of isolates mainly from clade C3 but also the three isolates from clade C4. In this period, no *Candida* species were reported in the sputum, but *S. aureus* was isolated. When the forced expiratory flow over the middle half of the forced vital capacity (FEF25–75%; thought to be indicative of the function of small/peripheral airways) was plotted against time, the same dynamics were observed but with greater magnitudes of functional loss, namely, 3.1%, 8.5%, and 2.3% declines per year for the three intervals (Fig. 8C). Here, a four-parameter logistic function fit to these FEF25−75% data provided greater explanatory power than a linear function or any other models (Fig. 8C legend). Although the sigmoidal models only modestly increase the fit to the data over a linear function that explains these spirometry dynamics well (increased *r*^2^ values of 0.05 for FEV_1_ and 0.03 for FEF25−75%), they also reduce the root mean square error (RMSE) by 15% and hence increase the predictive powers of both functions. This relative increase in the rate of decline in lung capacity coincides temporally with the rise of clade C3 as the dominant fraction of the *B. multivorans* infection. To summarize, both the phylogenetic composition of the *B. multivorans* isolates and the coinfecting pathogens correlated with the rate of decay in lung function.

## DISCUSSION

*B. multivorans* is the most commonly isolated *Burkholderia* species from chronic infections of the airways of CF patients, with an overall prevalence in the United States of 0.68% (43). However, our understanding of the traits required for bacterial colonization and persistence, as well as the molecular mechanisms underlying this adaptation, is limited. In this comprehensive study, we combined current genome sequencing technologies and phenotypic profiling of 22 longitudinally collected *B. multivorans* isolates from a single CF patient to gain new insight into the cause and consequences of pathogen diversification in chronic CF infections. We also associated these changes in the bacterial population with changes in the patient’s lung function. Not only did multiple evolutionary lineages coexist within this single patient for decades, multiple mutations whose phenotypes were almost certainly selected as adaptations evolved in relatively few loci and may have contributed to lung function decline.

Comparing the genomes of the 22 *B. multivorans* isolates revealed the steady accumulation of relatively subtle mutations over time, including 38 short insertions, 50 deletions, and 399 single-nucleotide changes. Similar evolutionary patterns were reported previously for *B. dolosa* (12, 13) and *P. aeruginosa* (5, 6, 44). The exception to this dynamic involved the final isolate from this study, BM22, which represents a lineage that acquired a frameshift mutation in *mutL* that led to an increased mutation rate and 196 new mutations. Mutators have often been described in *P. aeruginosa* isolates from CF patients (5, 45) and recently also for *B. cepacia* complex CF isolates (46). Isolate BM22, isolated in the year 2013, clusters with BM11 sampled in 2006, indicating that the *mutL* mutation might have occurred between 2006 and 2013. During the period of 2006 to 2013, the patient was treated with ceftazidime, tazobactam, and cloxacillin in 2008 and azithromycin, sulfamethoxazole, and trimethoprim in 2013, 8 months before the isolation of BM22, but neither these therapeutic interventions nor other coinfecting species correlated clearly with the *mutL* mutation. Mutator alleles likely encode no direct benefit but instead rise in frequency as traits linked to other adaptive mutations, but the large number of new mutations in this strain prevents inference of the conditions under which it evolved. Nevertheless, the side effect of mutator lineages is their accelerated evolutionary rate, making them inherently more difficult to control with antibiotics (47).

The phylogenetic analysis demonstrated that an initial infecting strain diversified into at least four major subpopulations (clades C1, C2, C3, and C4), each with its distinct genetic signature of mutations, with at least two of them coexisting at any time. Despite this diversity, clade C3 became the dominant lineage, not only because most isolates belong to this clade but also because they span a colonization period of 15 years. It is difficult to associate the success of the C3 population with any particular mutation, but the finding of multiple mutations in a sensor histidine kinase homologous to FixL in all isolates bears mentioning. A homolog of FixL has been shown to be involved in oxygen sensing and regulation of gene expression (23), and this gene was previously found to be the most polymorphic among 112 *B. dolosa* clinical isolates in a clonal outbreak of CF (12). Additional mutations found in clade C3 isolates, including those encoding a glucoamylase (also mutated in *B. dolosa* clinical isolates), a porin, and a type III secretion protein, and mutations in genes contributing to the metabolism of amino acids and lipids likely also influenced the success of this clade. These altered proteins may reflect selection on the basis of particular nutritional needs as well as on the basis of altered envelope biogenesis and host interactions. In total, these mutations associated with the diversification of clade C3, and shifts in other coinfecting species also coincided with the period of enhanced decline in lung function of the infected patient (Fig. 8). This evidence motivates the hypothesis that mutations in one or more of these genes were adaptive in this lung infection and may have accelerated the decline in patient lung function.

The mutations at the nodes of each of the clades provide overwhelming evidence of positive selection; only five synonymous mutations accompany 41 nonsynonymous mutations. In addition, most of the mutated genes at these nodes (some of which are also the most polymorphic) are involved mainly in transcription/signal transduction and lipid/amino acid metabolism. This role of regulatory genes in adaptation is echoed by the finding that common mutations in the *P. aeruginosa mucA* gene have extensive pleiotropic effects on gene expression and a general reduction of virulence factor expression (48). Here, the *B. multivorans rpfR*, *fixL*, and *ompR*-like genes encode proteins with regulatory functions and are likely candidates for governing global regulatory changes that influence host adaptation.

We also observed conspicuous changes in the physiology of these isolates, beginning with a reduced growth rate and biomass. Growth rate declines have also been observed for *P. aeruginosa* isolates obtained during chronic CF patient lung infections (48). Again, the temporal analysis of when these traits evolved suggests that coinciding mutations may explain them, including early mutations in genes encoding cell division protein FtsK and a DNA-binding regulatory protein from the YebC family. Which of these mutations is ultimately responsible and why a reduced growth rate may be adaptive are still unclear, though it might be a response to the new nutritional environment of CF patient sputum or a side effect of greater investment in adhesion and biofilm production.

Another trait under negative selection was the expression of the O-antigen repeats of LPS, a putative virulence factor in other *Burkholderia* organisms (26, 28). Except for BM1 and clade C4 isolates, all the other isolates displayed a rough phenotype from a lack of O-antigen repeats due to mutations in sugar epimerase enzymes. Conversely, *B. dolosa* exhibits a gain-of-function mutation that restores O-antigen presentation in isolates from several patients (12). It has also been shown that early isolates of the highly transmissible ET12 lineage of *B. cenocepacia* (J2315, BC7, and C5424) lack the O antigen, but the later isolate K56-2 expresses it (26). On one hand, the O antigen is known to interfere with adhesion to abiotic surfaces and bronchial epithelial cells and to stimulate the host immune response (27, 28), and its loss may be beneficial for cells entering a biofilm lifestyle like the one in CF patient lungs. On the other hand, it may inhibit phagocytosis (27), and its loss may enhance bacterial elimination. Notably, *P. aeruginosa* isolates from CF patients with chronic lung infections often have rough LPS, possibly allowing these bacteria to evade the host’s antibody-mediated immune response (49). Furthermore, a study of long-term adaptation in biofilm conditions *in vitro* by *B. cenocepacia* revealed mutations in LPS-associated loci, which implies that these mutations may enhance adherence or aggregation independently of host selection (24). Clearly, altered LPS biosynthesis can drive biofilm adaptation, and how this contributes to infections deserves further study.

Yet another metabolic trait under selection during this infection was lipid metabolism. Mutated genes included *fabD* (ACP *S*-malonyltransferase), acetyl-CoA carboxylase, and glycerol-3-phosphate acyltransferase. Remarkably, some strains acquired multiple nonsynonymous mutations in the same gene (*fabD*) (Table S3), suggesting that selection acted to continually refine this trait. The reason for this strong selection is unclear. Changes in lipid metabolism influence membrane composition and can alter the dynamics of membrane-bound signaling complexes, as described previously for the production of small colony variants (SCVs) (50). Further, one study showed a reduction of the degree of saturation of fatty acids produced by infecting *B. cenocepacia* organisms that coincided with the deterioration of pulmonary function in CF patients (51).

Still another evolved trait in some isolates was elevated c-di-GMP pools, which may be expected given its role in regulating adherence. As expected, increased c-di-GMP levels influenced other phenotypes known to be dependent on this signaling molecule, such as motility and biofilm formation (38). The decreased motility of isolates from clades C2, C3, and C4 correlates with increased biofilm formation and adhesion to CF lung epithelial cells, with the exception of clade C4 isolates that express O-antigen repeats in the outer membrane. These implicate loss of motility and increased biofilm production as two other important adaptations that occur either as a direct or an indirect response to selection in CF patient lungs.

Perhaps the best-studied trait associated with bacterial adaptation to CF patient lungs is increased resistance to antimicrobial agents. Several reports describe mutations in genes encoding efflux pumps and its regulators, DNA gyrase, among others, as under selection in *P. aeruginosa* (5, 52) and *B. dolosa* (12). We also observed a mutation in a gene encoding a component of an efflux pump and confirmed that isolates carrying that mutation had enhanced antibiotic resistance (Fig. 5A). All together, our results demonstrate that only a few genetic changes can fundamentally alter the physiology and fitness of bacteria evolving during chronic infections and that evolutionary adaptation can involve mutations of structural genes involved in specific traits, but also regulatory genes possibly affecting global gene expression. These mutations therefore shift the colonizing bacteria toward a “chronic infection phenotype” of slow growth, higher biofilm production, reduced motility, and greater antibiotic resistance (48).

Fortunately, the long-term *B. multivorans* infection of the CF patient under study has been mild, requiring only a few periods of antibiotic therapy and a single hospitalization. Nevertheless, a general decay of lung function occurred at various rates, as is commonly observed from a variety of contributing host and microbiological factors (53). Data available for this patient suggest a higher diversity of the bacterial community before the onset of *B. multivorans* infection, since 2 to 3 years following the first isolate, *P. aeruginosa* was eradicated and only *Candida* and *S. aureus* were identified as coinfecting organisms. Supporting this observation, a recent report shows that CF-associated pathogens such as *Burkholderia*, *Pseudomonas*, *Stenotrophomonas*, and *Achromobacter*, have a strong tendency to dominate the bacterial community and that lower community diversity correlates with worse lung function (54). Therefore, it is not surprising that the period of accelerated lung function decline was associated with the presence of *B. multivorans* isolates displaying phenotypes usually attributed to chronic colonization, such as lower motility, increased biofilm formation, loss of O-antigen repeats, and antibiotic resistance, as well as diminished overall bacterial diversity. This was not the only change in the lung microbial community at this time, and it is worthy of mention that *Candida* was often isolated in this period, which is consistent with recent observations that yeasts/fungi such as *Candida albicans* contribute to lung function decline (55, 56). Whether the evolution of the *B. multivorans* population was shaped by interactions with other species and/or whether microbial interactions influenced the deterioration of lung function remain important open questions.

We have presented a detailed analysis of the temporal sequence of all of the genetic and many phenotypic changes that lead to the successful establishment of a *B. multivorans* chronic CF patient lung infection. The evolutionary dynamics revealed an initial 2- to 3-year period of adaptation to the CF patient airways, with extensive phenotypic changes and genetic diversification followed by a long period of more-limited phenotypic changes despite relatively constant genomic evolution (Fig. 1). Although *B. multivorans* adaptation to the CF lung seems to follow patterns of mutation similar to those described for *B. dolosa* and even *P. aeruginosa* (5, 12, 13), more isolates of *B. multivorans* from chronic infections should be analyzed to understand the success of this species among bacterial pathogens of CF patient airways. In addition, studying how *Burkholderia* species interact with the microbiomes of patients and correlate with disease status will provide further insights into the biology and decline in function of CF patient lungs.

In summary, we developed a detailed genome-wide model of evolution within a diverse population of *B. multivorans* isolates over more than 2 decades of chronic infection. This analysis clearly identified genes that are under strong positive selection, including mutations that produced long-lived lineages (e.g., *rpfR*) and multiple independent mutations in the same gene that recurrently altered key functions (e.g., *fixL*, *plsX* gene, *hfq*, an *ompR*-like gene, and a *fis*-like gene). Remarkably, 10 loci were found with three or more independent mutations irrespective of the genetic background of the isolates. As these genes are not especially prone to mutation (22), this provides strong evidence that their predicted phenotypes are exceptionally important for successful persistence in the CF patient lung environment. We also assayed a broad array of phenotypes that were either directly or strongly associated with the predicted functions of these driver mutations (e.g., elevated c-di-GMP levels, increased antibiotic resistance, and altered O antigen). Collectively, these strongly selected traits represent a panel that should enable genome-based diagnostics for more-personalized treatment of airway infections in patients with CF and perhaps other chronic infections. This knowledge also points to potential therapeutic targets and can direct efforts for the development of effective treatments against *B. cepacia* complex bacteria.

## MATERIALS AND METHODS

### Bacterial strains and growth conditions.

The 22 investigated bacterial isolates (see Table S1 in the supplemental material) consist of a single clone of *Burkholderia multivorans* sampled from a patient attending a CF clinic in Vancouver, Canada, spanning the period between 1993 and 2013. This patient was chosen based on the fact that the initial isolates and most of the remaining ones were mucoid, but three isolates collected in the years 1997, 2006, and 2013 were nonmucoid and thus examples of the mucoid-to-nonmucoid transition (14). *Burkholderia* isolates were grown in LB or in extracellular polymeric substance (EPS)-producing salt-mannitol (SM) medium (16) at 37°C and were stored at −80°C with 30% glycerol. *E. coli* strains were grown in LB at 37°C. When it was necessary to maintain the selective pressure, growth media were supplemented with antibiotics at the following concentrations: for *B. multivorans*, chloramphenicol at 200 μg ml^−1^, and for *E. coli*, chloramphenicol at 25 μg ml^−1^ and kanamycin at 50 μg ml^−1^.

### Genome sequencing, assembly, and annotation.

Genomic DNA from the *B. multivorans* clinical isolates was extracted and purified using the DNeasy blood and tissue kit (Qiagen). DNA samples were processed according to Illumina’s instructions for generating paired-end libraries. Paired-end libraries of *B. multivorans* D2095 (other designations: VC13401; BM11) and D2214 (VC13534; BM12) were sequenced using the Illumina HiSeq2000 platform at Fasteris SA (Switzerland), the mate-pair library of *B. multivorans* D2095 and the paired-end library of *B. multivorans* VC5602 (BM1) were sequenced using an Illumina HiSeq2500 at BaseClear BV (Netherlands), and paired-end libraries of the remaining 19 *B. multivorans* isolates were sequenced using an Illumina MiSeq system at the Instituto Gulbenkian de Ciência (Portugal).

*B. multivorans* D2095 (BM11) was used as the reference genome, and sequencing and *de novo* assembly of this genome were described previously (BioProject accession no. PRJNA239698) (57). In more detail, raw paired-end reads (two 100-bp reads with an insert size of around 300 bp and ~1,000-fold coverage), and an extra paired-end library of D2095 (BM11) obtained with an Illumina HiSeq2000 (~80-fold coverage) and two 50-bp mate-pair reads with an insert size of 5 kb (~70-fold coverage) were corrected using Quake v.0.3 (58). These Quake-corrected data sets were processed using the fastq-mcf tool (59) and filtered based on Phred quality scores. Adapter contamination and low-quality and ambiguous nucleotides were trimmed from the remaining reads. The overall quality assessment of each corrected data set was carried out using FastQC (60). Paired-end reads from the HiSeq2000 were *de novo* assembled using Edena v.3.131028 (61) and SPAdes v.3.0.0 (62). The quality of assemblies obtained by using different parametrizations was estimated by assessing the number of single-nucleotide variants (SNVs) and indels when the filtered HiSeq2000 paired-end reads were mapped against the *de novo* assemblies with Bowtie2 (63), SAMtools v.0.1.19 (64), and SNVer 0.5.2 (65) for format file conversion and single-nucleotide polymorphism (SNP) detection, respectively. The assemblies from Edena and Spades assemblers were merged using GAM-NGS (66), followed by automated improvement using Pilon v.1.6 (67). The three filtered data sets (two paired-end libraries and one mate-pair library) from the Illumina HiSeq2000 were used for scaffolding by SSPACE v.2.3 (68), gap closure by GapFiller v.1.5 (69), and GapCloser v.1.12 (70). The final draft genome sequence of *B. multivorans* D2095 was obtained after automatic improvement using Pilon and manual curation. Genome annotation was carried out using the National Center for Biotechnology Information Prokaryotic Genomes Automatic Annotation Pipeline (NCBI PGAAP), v.2.9.

### Detection of SNP and indel mutations.

Raw paired-end reads from 21 *B. multivorans* isolates generated with an Illumina MiSeq were filtered based on Phred quality scores. Adapter contamination and ambiguous nucleotides were trimmed off using the fastq-mcf tool (59). Only reads with Phred scores higher than 30 (99.9% accuracy) were considered for subsequent analysis, yielding a mean of 92% of the genomes with ≥20-fold coverage (see Table S6 in the supplemental material). Filtered paired-end data sets were mapped against the reference draft genome sequence of *B. multivorans* D2095 (BM11) using BWA-MEM of BWA v.0.7.10 (71, 72) and NovoAlign v.3.02.07 (Novocraft; website last accessed 6 June 2015) to determine a first list of putative mutations. Each SNP was analyzed according to the Phred score of SNP calls, the SNP calls on forward and reverse reads, and the read depth of the inspected region. Only SNPs and indels detected in alignments using both BWA-MEM and NovoAlign and occurring in at least three forward and reverse reads considered high-quality SNPs were further analyzed. In parallel, SNVer 0.5.3 (65) was used to call variants and to produce .vcf files for both BWA-MEM- and NovoAlign-generated assemblies. A frequency of 90% was considered to call an SNP real, and reads with coverage of lower than 20-fold were carefully evaluated by visual inspection using Geneious v.6.1.8 (73), with which the existence of other variable nucleotides in the same region was considered a strong indicator of error. The SNPs and indels shown in Table S3 were normalized against the genome sequence of the first isolate, VC5602 (BM1).

10.1128/mSystems.00029-16.6 Table S6  Sequencing statistics of each *B. multivorans* isolate genome. Download Table S6, DOCX file, 0.04 MB. Copyright © 2016 Silva et al. 2016 Silva et al. This content is distributed under the terms of the Creative Commons Attribution 4.0 International license.

### Phylogenetic analysis.

To infer the phylogeny of the 22 *B. multivorans* isolates, SNPs and indel mutations (337 positions) were concatenated separately for each isolate (Table S3) and analyzed as two distinct substitution models (substitutions: Tamura-Nei and indel RS07) with the Bali-Phy package (21). Following multiple runs with a Bayesian Markov chain Monte Carlo (MCMC) approach, a consensus maximum *a posteriori* tree was generated. This approach is explicitly aware of the distinct mutational processes and spectra of SNPs and indels.

### Distribution of mutations expected for neutral evolution.

To determine the distribution of mutations expected under a model of neutral evolution, we randomly generated 192 mutations in the 5,809 genes of the *B. multivorans* D2095 genome and counted the distribution of the number of mutations obtained per gene. This was repeated 1,000 times, and mutations occurring in the last mutator isolate, BM22, were excluded. A permutation test (R v.3.2.2, Biostrings package v.2.36.3) was then used to determine the probability that any given gene is mutated any number of times.

### Molecular cloning and in *trans* complementation experiments.

The region upstream of *BMD20_17715* and the coding sequences of *BMD20_17715* (*wbiF*) and *BMD20_17710* (*wbiG*) were amplified from *B. multivorans* BM1 genomic DNA by PCR using the primers P1 (CTAGGGTACCGTGCCGACCTATCCTGCC [italics indicate restriction sites]) and P2 (CGTCTAGATATCGGGAGATGCATGTCG). The amplification conditions were 2 min at 95°C, 30 cycles of 95°C for 45 s, 59°C for 30 s, and 72°C for 2 min, and a final extension of 7 min at 72°C. The resulting 1,951-bp product was digested with KpnI and XbaI and cloned in the same restriction sites of pBBR1MCS. After *E. coli* DH5α transformation and clone selection, the plasmid obtained was named pLM015-12 (see Table S1 in the supplemental material). Plasmid pLM015-12 was mobilized into *B. multivorans* isolates by triparental mating using *E. coli* DH5α(pRK2013) as a helper strain (74). Exconjugants from the transfer of pLM015-12 were selected on LB agar plates supplemented with 200 µg of chloramphenicol ml^−1^ and 20 µg of gentamicin ml^−1^.

### Growth rate and doubling time determination.

Isolates were grown overnight in liquid synthetic cystic fibrosis medium (SCFM) (75) at 37°C. A volume was then centrifuged (1 min, 10,000 × *g*), and the pellet was washed with saline solution (0.9% NaCl) and used to inoculate a microplate with 200 µl of SCFM in each well (5 wells for each isolate; 12 isolates per plate), generating an initial optical density at 640 nm (OD_640_) of 0.05. Microplates were incubated in a microplate reader at 37°C with shaking for 5 s every 30 min, and growth rates were measured by monitoring the OD_640_ for 22 h. Growth rates were calculated from the exponential phase of growth from at least three independent experiments. Statistical analysis was carried out using Excel with QI Macros 2016. One-way analysis of variance (ANOVA) and Tukey’s honestly significant difference (HSD) multiple-comparison test for unequal group sample sizes were performed to determine statistically significant differences. Differences were considered statistically significant if the *P* value was lower than 0.05.

### Analysis of lipopolysaccharide and exopolysaccharide.

LPS was extracted as described previously (76), with minor modifications. Briefly, cells from overnight plate cultures were suspended in a lysis buffer containing DNase and incubated for 30 min at 37°C, followed by proteinase K digestion for 4 h at 60°C. Then, LPS was purified by hot phenol extraction and a subsequent extraction of the aqueous phase with ether. LPS samples were resolved by electrophoresis in 16% polyacrylamide gels with a Tricine-SDS system (77) and silver stained.

The amount of exopolysaccharide being produced was evaluated based on the dry weight of the ethanol-precipitated polysaccharide recovered from 100-ml cultures grown in SM medium for 6 days at 37°C with orbital agitation (78). Data are based on results from duplicate flasks from three independent experiments.

### Antimicrobial susceptibility.

The agar disc diffusion method (79) was used to assess isolate susceptibility against paper discs containing piperacillin (75 µg) plus tazobactam (10 µg). The discs were applied onto the surfaces of Muller-Hinton (Difco Laboratories) agar plates previously inoculated with 100 µl of a suspension at an OD_640_ of 0.1 prepared from exponential-phase cells growing on LB medium. The diameter of the growth inhibition zone was measured after 24 h of incubation at 37°C. Three experiments, each with three discs per isolate, were performed. Statistical analysis was conducted by ANOVA and Tukey’s HSD multiple-comparison test.

### Motility.

The swimming motility of all isolates was tested on 0.3% (wt/vol) agarose supplemented with 1% tryptone and 0.5% NaCl. Plates were spot inoculated with a 3-µl drop of a culture at an OD_640_ of 1.0 and were incubated for 24 h at 37°C. The motility zones of three replicates from three independent experiments were measured. Statistical analysis was conducted by ANOVA and Tukey’s HSD multiple-comparison test.

### Measurement of c-di-GMP levels.

For c-di-GMP extraction, *B. multivorans* isolates were cultured in triplicate in SM medium for 5 h. Cells from a 40-ml culture were harvested by centrifugation (10,000 × *g*), and pelleted cells were resuspended in extraction buffer (40% methanol, 40% acetonitrile, 0.1 N formic acid). This mixture was incubated at −20°C for 30 min, after which insoluble cell debris were precipitated by centrifugation at 14,000 × *g* for 5 min (80). A total volume of 200 µl of the supernatants was neutralized with 8 µl of 15% (wt/vol) NH_4_HCO_3_. To measure the c-di-GMP of the lysates, 10 µl of each sample was analyzed using liquid chromatography-tandem mass spectrometry (LC-MS/MS) as described previously (80). Concentrations are reported as nanomolar concentrations of c-di-GMP, assuming cylinder-shaped bacteria with a volume of 8.3 × 10^−16^ liters and taking into account the number of CFU in each sample. Statistical analysis was conducted by ANOVA, followed by Tukey’s HSD multiple-comparison test.

### Biofilm formation.

Biofilm formation assays were performed as described previously (81). Bacteria were grown in LB medium at 37°C to mid-exponential phase and diluted to an OD_640_ of 0.05, and 200-µl samples of these cell suspensions were used to inoculate 96-well polystyrene microtiter plates. Plates were incubated at 37°C statically for 48 h, after which the wells were washed three times with 0.9% (wt/vol) NaCl. The biofilm was stained with 1% (wt/vol) crystal violet solution for 20 min, followed by dye solubilization with ethanol and measurement of the solution’s *A*_590_ in a microplate reader. Results are the means of results from six replicates of five independent experiments. ANOVA and Tukey’s HSD multiple-comparison test were performed to determine statistically significant differences.

### Adhesion to epithelial cells.

The *B. multivorans* isolates under study were examined for adhesion to the bronchial epithelial cell line CFBE41o^−^, derived from a patient homozygous for the cystic fibrosis transmembrane conductance regulator *ΔF508* mutation (82). Host cell attachment was performed as described previously (83). Briefly, bacterial strains grown in SM medium to an OD_640_ of 0.6 (log phase) were used to infect epithelial cells at a multiplicity of infection (MOI) of 10 (10 bacterial cells to 1 epithelial cell). Bacteria were applied to a 24-well plate previously seeded with CFBE41o^−^ cells in minimal essential medium-supplemented medium, and the plates were centrifuged at 700 × *g* for 5 min. The plates were incubated for 30 min at 37°C in an atmosphere of 5% CO_2_. Each well was then washed three times with phosphate-buffered saline (PBS) to remove unbound bacteria, and cells were lysed with lysis buffer (0.01 M PBS, 20 mM EDTA, 0.5% [vol/vol] Triton X-100; pH 7.4) for 30 min at 4°C. Serial dilutions were plated onto LB agar, and adhesion was quantified by determining CFU counts after 48 h of incubation at 37°C. Duplicates with each strain were performed per assay, and the results presented were obtained from three independent experiments. Results are expressed as the percentage of adhesion, which was calculated as the number of CFU recovered divided by the number CFU applied to the epithelial cells multiplied by 100. Results are the mean values from two replicates from three independent experiments. ANOVA followed by Tukey’s HSD multiple-comparison test was performed.

### Ethics statement.

The bacterial samples used are from an anonymous patient, and none of the samples was taken specifically for this study. The patient’s clinical data were collected as part of a previously published chart review study, which was approved by the University of British Columbia’s Research Ethics Boards (approval H07-01396).

### Nucleotide sequence accession numbers.

The DNA sequence reads for assemblies of the several genomes are available in the EMBL’s European Nucleotide Archive (ENA) under accession number PRJEB13383. Fully assembled and annotated genomes of *B. multivorans* VC13401 (D2095) and VC13534 (D2214) are available in the NCBI database under accession numbers PRJNA239698 and PRJNA239704, respectively. Alignment and SNP call files are in figshare (https://figshare.com/s/58adae59e3ebd57550da).
