# Supplementary material for: Long-Term Evolution of Burkholderia multivorans during a Chronic Cystic Fibrosis Infection Reveals Shifting Forces of Selection
Source: mSystems. 2016 May 24;1(3):e00029-16. doi: 10.1128/mSystems.00029-16 (PMC5069766; doi:10.1128/mSystems.00029-16)
Supplement: Table S1 [file sys003162026st1.docx]

**Table S1** **Bacterial strains used in this study.**

| Bacterial strain | Relevant characteristics*^a^* | Source or reference^c^ |
| --- | --- | --- |
| *Burkholderia multivorans^b^* |  |  |
| VC5602 (BM1) | CF clinical isolate; Isolation: 30 November 1993; from sputum (formerly: C5568) | CBCCRRR |
| VC7102 (BM2) | CF clinical isolate, Vancouver; Isolation: 26 May 1995; from throat (formerly: C6558) | CBCCRRR |
| VC7731 (BM3) | CF clinical isolate; Isolation: 14 June 1996; from sputum (formerly: C7148) | CBCCRRR |
| VC7732 (BM4) | CF clinical isolate; Isolation: 14 June 1996; from sputum (formerly: C7149) | CBCCRRR |
| VC8270 (BM5) | CF clinical isolate; Isolation: 06 June 1997; from sputum (formerly: C7637) | CBCCRRR |
| VC8999 (BM6) | CF clinical isolate; Isolation: 20 June 1998; from sputum (formerly: C8179) | CBCCRRR |
| VC10371 (BM7) | CF clinical isolate; Isolation: 23 September 2000; from sputum (formerly: C9326) | CBCCRRR |
| VC11265 (BM8) | CF clinical isolate; Isolation: 29 March 2002; from throat (formerly: D0089) | CBCCRRR |
| VC13059 (BM9) | CF clinical isolate; Isolation: 03 October 2005; from throat (formerly: D1782) | CBCCRRR |
| VC13400 (BM10) | CF clinical isolate; Isolation: 01 June 2006; from throat (formerly: D2094) | CBCCRRR |
| VC13401 (BM11) | CF clinical isolate; Isolation: 01 June 2006; from throat (formerly: D2095) | CBCCRRR |
| VC13534 (BM12) | CF clinical isolate; Isolation: 09 November 2006; from sputum (formerly: D2214) | CBCCRRR |
| VC14024 (BM13) | CF clinical isolate; Isolation: 27 October 2007; from throat | CBCCRRR |
| VC14765 (BM14) | CF clinical isolate; Isolation: 07 May 2009; from sputum | CBCCRRR |
| VC15201 (BM15) | CF clinical isolate; Isolation: 19 April 2010; from throat | CBCCRRR |
| VC15656 (BM16) | CF clinical isolate; Isolation: 20 June 2011; from sputum | CBCCRRR |
| VC15765 (BM17) | CF clinical isolate; Isolation: 23 September 2011; from sputum | CBCCRRR |
| VC15766 (BM18) | CF clinical isolate; Isolation: 23 September 2011; from sputum | CBCCRRR |
| VC16144 (BM19) | CF clinical isolate; Isolation: 31 May 2012; from sputum | CBCCRRR |
| VC16557 (BM20) | CF clinical isolate; Isolation: 28 February 2013; from sputum | CBCCRRR |
| VC16559 (BM21) | CF clinical isolate; Isolation: 28 February 2013; from sputum | CBCCRRR |
| VC16929 (BM22) | CF clinical isolate; Isolation: 27 October 2013 | CBCCRRR |
|  |  |  |
| *Escherichia coli* |  |  |
| DH5α | K-12 Φ80d *lacZ*_M15 *endA1 recA1 hsdR17* (r_K_^–^m_K_^–^) *supE44 thi-1 gyrA96 relA1* Δ*(lacZYA-argF)* | Invitrogen |
|  |  |  |
| Plasmids |  |  |
| pBBR1MCS | 4,717-bp broad-host-range cloning vector, Cm^r^ | (1) |
| pRK2013 | Tra^+^ Mob^+^ (RK2) Km::Tn*7* ColEl origin, helper plasmid, Km^r^ | (2) |
| pLM015-12 | pBBR1MCS derivative containing 2,141-bp KpnI/XbaI fragment with the region upstream of *BMD20_17715* and the coding regions of *BMD20_17715* and *BMD20_17710* | This work |

*^a^*Abbreviations: Cm^r^, chloramphenicol resistance; Km^r^, kanamycin resistance. *^b^*For simplicity, clinical isolates identification was shortned; ^c^CBCCRRR, Canadian *Burkholderia cepacia* complex research and referral repository.

1. **Kovach ME**, **Phillips RW**, **Elzer PH**, **Roop 2nd RM**, **Peterson KM**. 1994. pBBR1MCS: a broad-host-range cloning vector. Biotechniques **16**:800–802.

2. **Figurski DH**, **Helinski DR**. 1979. Replication of an origin-containing derivative of plasmid RK2 dependent on a plasmid function provided in trans. Proc Natl Acad Sci U S A **76**:1648–1652.
