# Supplementary material for: Long-Term Evolution of Burkholderia multivorans during a Chronic Cystic Fibrosis Infection Reveals Shifting Forces of Selection
Source: mSystems. 2016 May 24;1(3):e00029-16. doi: 10.1128/mSystems.00029-16 (PMC5069766; doi:10.1128/mSystems.00029-16)
Supplement: Table S2 [file sys003162026st2.docx]

**Table S2. Different species isolated from this CF patient airways.**

| Date | Species | # identified colonies | Stored isolates |
| --- | --- | --- | --- |
| 07-Mar-1989 | *Pseudomonas aeruginosa*  *Staphylococcus aureus* | 1  1 |  |
| 06-Mar-1990 | *Pseudomonas aeruginosa*  *Staphylococcus aureus* | 1  1 |  |
| 12-Mar-1991 | *Pseudomonas aeruginosa*  *Staphylococcus aureus* | 1  2 |  |
| 07-Jan-1992 | *Staphylococcus aureus* | 2 |  |
| 05-May-1992 | *Pseudomonas aeruginosa*  *Staphylococcus aureus* | 1  2 |  |
| 11-Aug-1992 | *Pseudomonas aeruginosa*  *Staphylococcus aureus* | 1  2 |  |
| 08-Dec-1992 | *Pseudomonas aeruginosa*  *Staphylococcus aureus* | 1  2 |  |
| 09-Mar-1993 | *Haemophilus influenzae* | 1 |  |
| 08-Jul-1993 | *Staphylococcus aureus* | 1 |  |
| 30-Nov-1993 | *Staphylococcus aureus*  *Burkholderia multivorans* | 2  1 | BM1 |
| 22-Feb-1994 | *Staphylococcus aureus* | 1 |  |
| 08-Nov-1994 | *Staphylococcus aureus* | 1 |  |
| 16-May-1995 | *Pseudomonas aeruginosa*  *Staphylococcus aureus*  *Burkholderia multivorans* | 1  1  1 | BM2 |
| 14-Jun-1996 | *Pseudomonas aeruginosa* | 2 |  |
| 12-Dec-1997 | *Burkholderia multivorans*  *Staphylococcus aureus* | 1  1 |  |
| 20-Jun-1998 | *Burkholderia multivorans*  *Staphylococcus aureus* | 2  1 | BM6 |
| 25-Jun-1999 | *Staphylococcus aureus*  *Candida* | 1  1 |  |
| 23-Sep-2000 | *Burkholderia multivorans*  *Staphylococcus aureus* | 4  1 | BM7 |
| 29-Mar-2002 | *Burkholderia multivorans* | 1 | BM8 |
| 09-Jul-2003 | *Burkholderia multivorans* | 2 |  |
| 14-Nov-2003 | *Burkholderia multivorans*  *Candida* | 2  1 |  |
| 03-Oct-2005 | *Burkholderia multivorans*  *Candida* | 3  1 | BM9 |
| 31-May-2006 | *Burkholderia multivorans*  *Candida* | 2  1 | BM10; BM11 |
| 08-Nov-2006 | *Burkholderia multivorans* | 2 | BM12 |
| 29-Oct-2007 | *Burkholderia multivorans* | 3 | BM13 |
| 05-May-2008 | *Burkholderia multivorans* | 2 |  |
| 06-May-2009 | *Burkholderia multivorans*  *Staphylococcus aureus* | 2  1 | BM14 |
| 16-Sep-2009 | *Burkholderia multivorans* | 1 |  |
| 19-Apr-2009 | *Burkholderia multivorans*  *Staphylococcus aureus* | 3  1 | BM15 |
| 22-Nov-2010 | *Burkholderia multivorans* | 2 |  |
| 28-Jun-2011 | *Burkholderia multivorans* | 2 | BM16 |
| 28-Sep-2011 | *Burkholderia multivorans*  *Staphylococcus aureus* | 4  1 | BM17; BM18 |
| 15-Feb-2012 | *Burkholderia multivorans*  *Staphylococcus aureus* | 3  1 |  |
| 31-May-2012 | *Burkholderia multivorans*  *Staphylococcus aureus* | 3  1 | BM19 |
| 23-Oct-2012 | *Burkholderia multivorans* | 2 |  |
| 28-Feb-2013 | *Burkholderia multivorans*  *Staphylococcus aureus* | 3  1 | BM20; BM21 |
| 27-Oct-2013 | *Burkholderia multivorans* | 1 | BM22 |
