# Supplementary figures and images for: Long-Term Evolution of Burkholderia multivorans during a Chronic Cystic Fibrosis Infection Reveals Shifting Forces of Selection
Source: mSystems. 2016 May 24;1(3):e00029-16. doi: 10.1128/mSystems.00029-16 (PMC5069766; doi:10.1128/mSystems.00029-16)

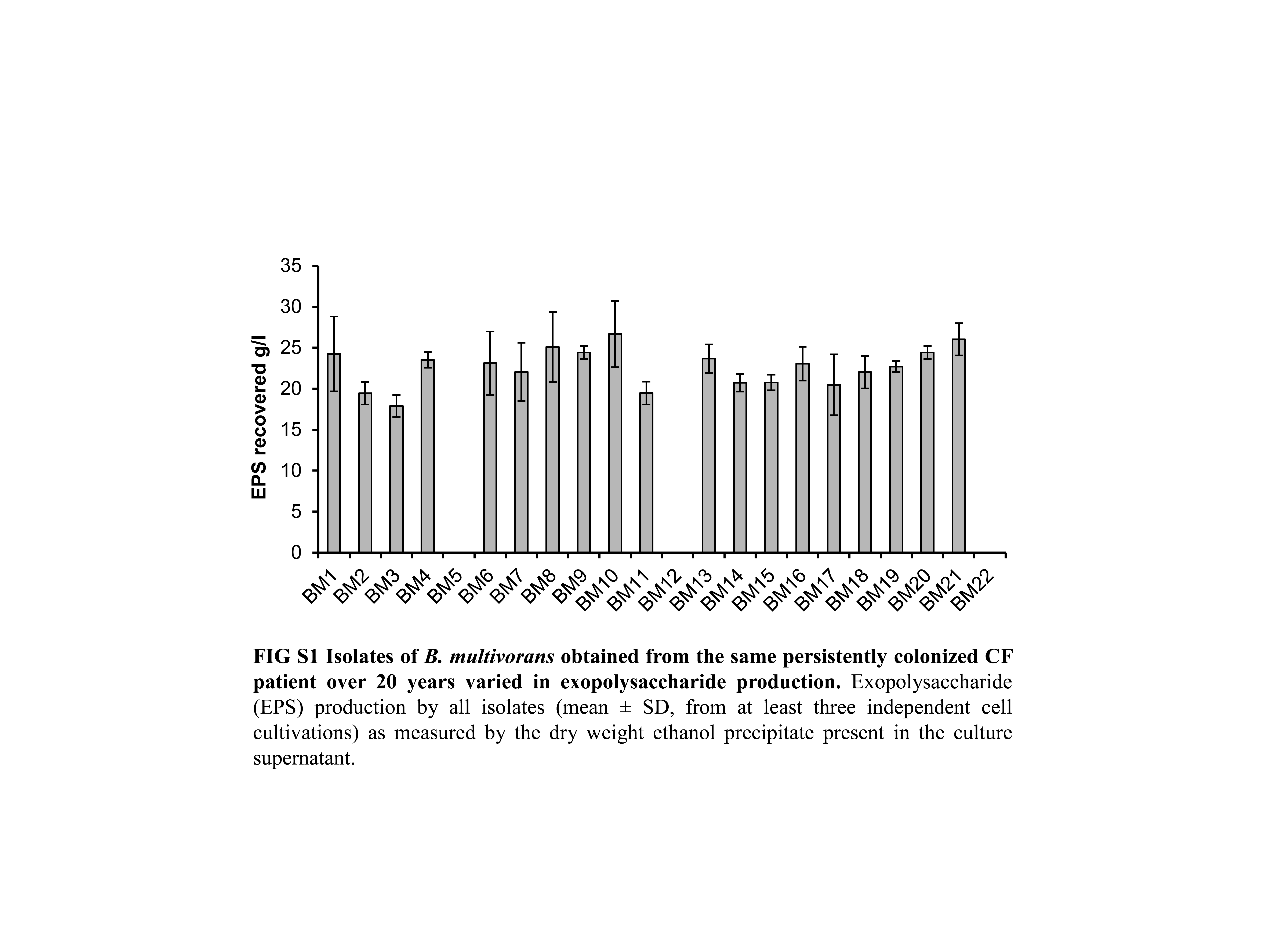

Supplement: Figure S1 [file sys003162026sf7.tif]

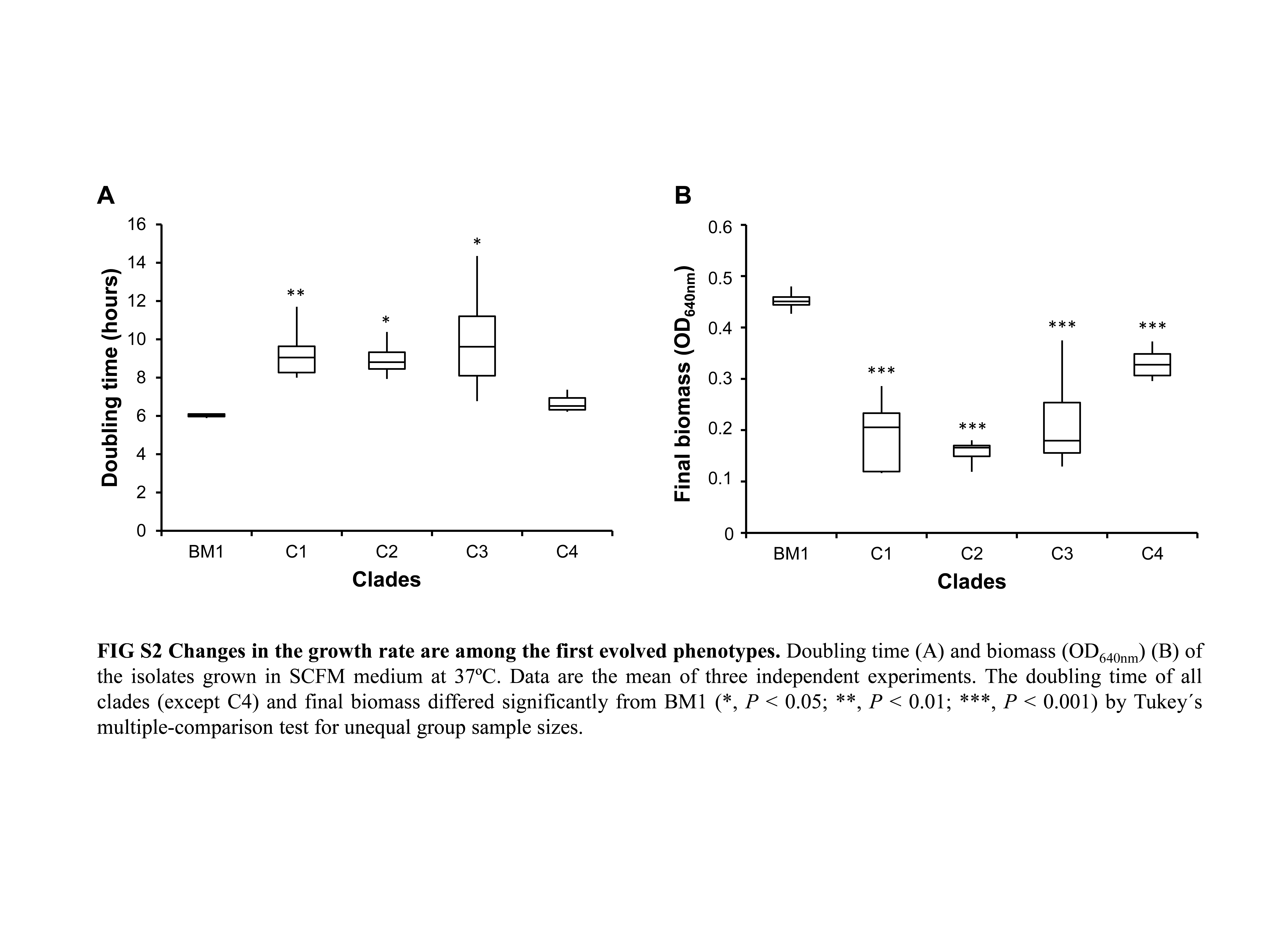

Supplement: Figure S2 [file sys003162026sf8.tif]

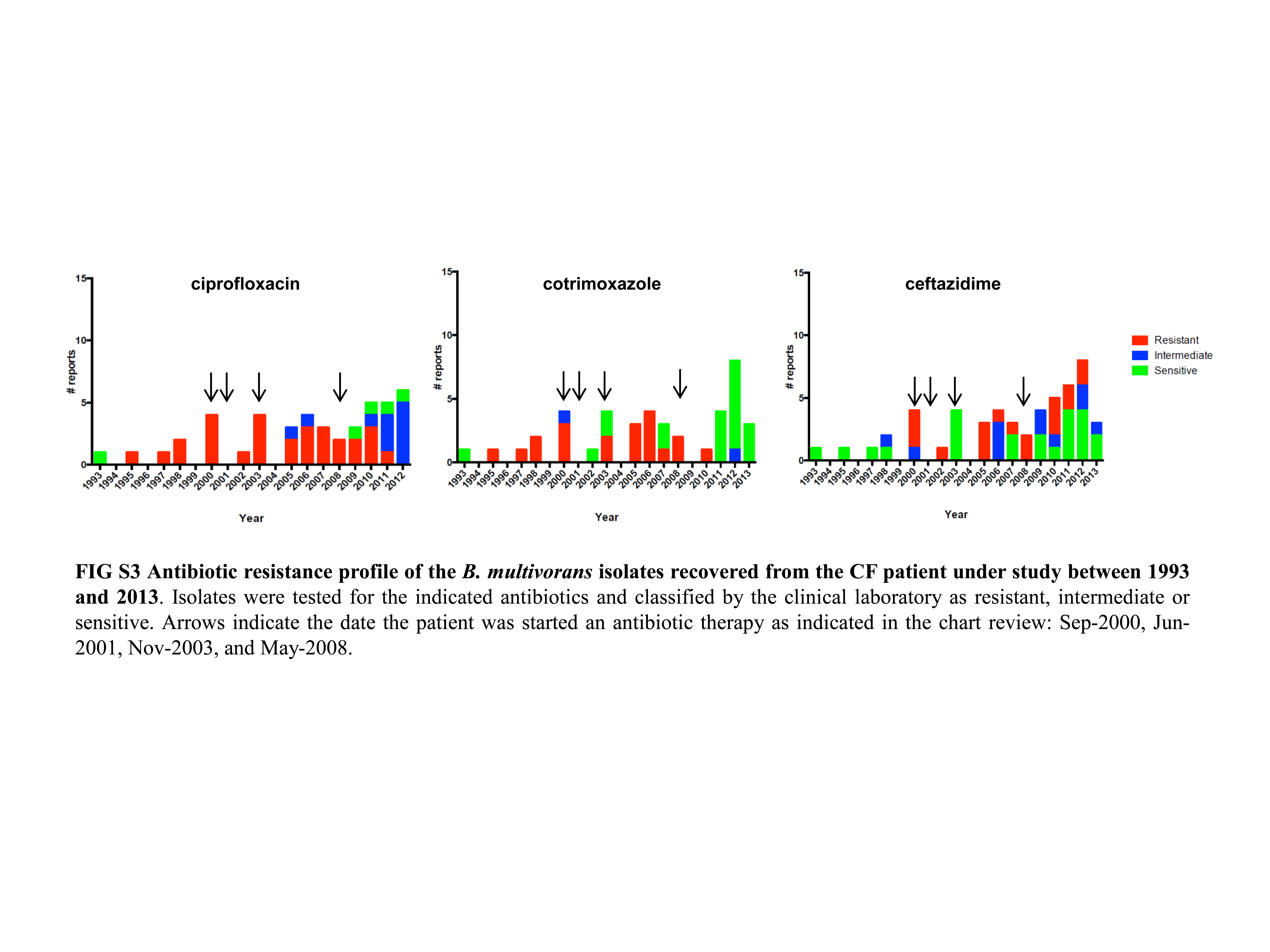

Supplement: Figure S3 [file sys003162026sf9.tif]

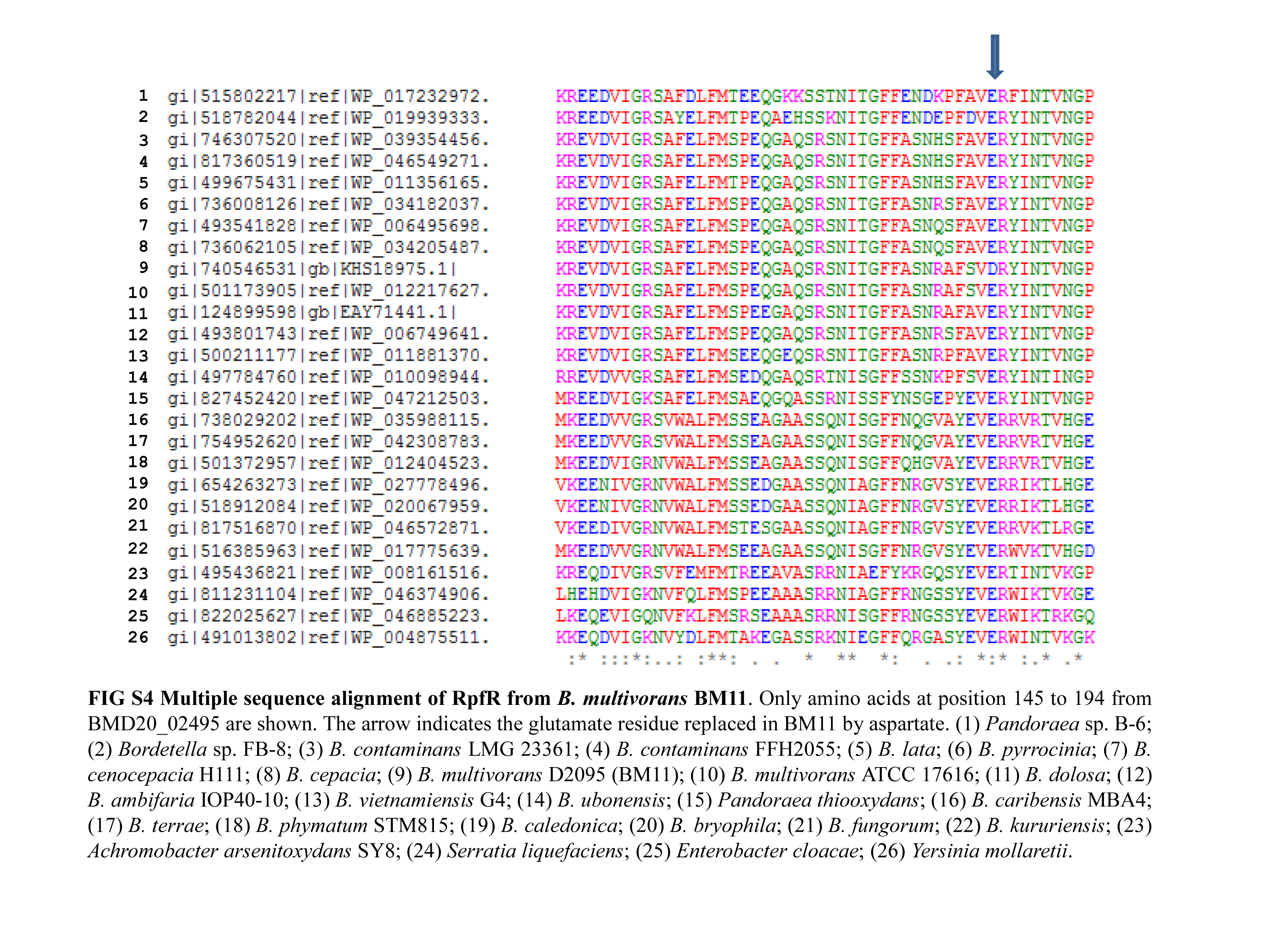

Supplement: Figure S4 [file sys003162026sf10.tif]
