# Supplementary material for: Long-Term Evolution of Burkholderia multivorans during a Chronic Cystic Fibrosis Infection Reveals Shifting Forces of Selection
Source: mSystems. 2016 May 24;1(3):e00029-16. doi: 10.1128/mSystems.00029-16 (PMC5069766; doi:10.1128/mSystems.00029-16)
Supplement: Table S5 [file sys003162026st5.docx]

**Table S5.** **Quantitative real-time RT-PCR analysis of BMD20_11660 gene of *B.* *multivorans* isolates relative to the first isolate, BM1.**

| Isolate | Phylogenetic clade | Real-time fold-change ± SD |
| --- | --- | --- |
| BM2 | C1 | 1.6 ± 0.4 |
| BM9 | C3 | 14.6 ± 2.7 |
| BM10 | C4 | 12.9 ± 3.6 |
| BM11 | C3 | 8.7 ± 1.7 |

Quantitative real-time RT-PCR (qRT-PCR)

Expression of the *ompR*-like gene (*BMD20_11660*) was quantified by qRT-PCR. For total RNA extraction, bacterial cells were grown in 100 ml SM medium, in triplicates, at 37ºC, 250 rpm. After 10 hours, 2-ml samples of each culture were resuspended in RNAprotect bacteria reagent (Qiagen) and total RNA was extracted using RNeasy MidiKit (Qiagen) following manufacturer’s instructions. Total RNA was used in reverse transcription reaction with TaqMan Reverse Transcription Reagents (Applied Biosystems). qRT-PCR amplification of *BMD20_11660* (ompR-RT-PCR-Fw, CGAGCAAGGCTTCAACGTCTA and ompR-RT-PCR-Rev, CGCACCCAGAGTTTGTTCATC) and *proC* (proC-RT-PCR-Fw, GTCGGCGAGATCGTAGGTT and proC-RT-PCT-Rev, CTGCAGCGCTTCGATGAAA) as housekeeping control was performed in a Thermocycler 7500 (Applied Biosystems). Relative quantification of gene expression by qRT-PCR was determined using the ∆∆C_T_ method (1).

1. **Pfaffl MW**. 2001. A new mathematical model for relative quantification in real-time RT-PCR. Nucleic Acids Res **29**:e45.
