# Supplementary material for: Long-Term Evolution of Burkholderia multivorans during a Chronic Cystic Fibrosis Infection Reveals Shifting Forces of Selection
Source: mSystems. 2016 May 24;1(3):e00029-16. doi: 10.1128/mSystems.00029-16 (PMC5069766; doi:10.1128/mSystems.00029-16)
Supplement: Table S6 [file sys003162026st6.docx]

**Table S6.** **Sequencing statistics of each *B. multivorans* isolate genome.**

| **Isolate** | **Nº of paired-end reads** | **Average coverage (fold)** | **% genome with >20x coverage** | **SNPs/indels** |
| --- | --- | --- | --- | --- |
| BM1 | 6,747,676 | 91* | 98.4 | 0/0 |
| BM2 | 966,142 | 29 | 83.3 | 8/3 |
| BM3 | 1,537,560 | 45 | 97.8 | 6/3 |
| BM4 | 1,172,272 | 34 | 80.8 | 20/3 |
| BM5 | 1,538,096 | 45 | 97.4 | 17/4 |
| BM6 | 1,499,052 | 44 | 97.3 | 36/7 |
| BM7 | 1,509,350 | 44 | 97.7 | 30/5 |
| BM8 | 1,404,218 | 41 | 96.3 | 37/11 |
| BM9 | 1,082,614 | 32 | 87.7 | 45/16 |
| BM10 | 1,014,464 | 30 | 87.2 | 38/7 |
| BM11 | 71,916,352 | 971* | 100.0 | 45/9 |
| BM12 | 56,668,592 | 765* | 97.0 | 48/11 |
| BM13 | 1,166,698 | 33 | 88.8 | 36/6 |
| BM14 | 1,346,202 | 38 | 94.5 | 50/12 |
| BM15 | 1,148,622 | 32 | 85.3 | 39/9 |
| BM16 | 1,551590 | 44 | 96.9 | 46/10 |
| BM17 | 1,023626 | 29 | 81.4 | 53/14 |
| BM18 | 1,685,874 | 47 | 97.4 | 54/17 |
| BM19 | 1,415,654 | 39 | 93.2 | 53/14 |
| BM20 | 1,806,134 | 50 | 97.6 | 70/16 |
| BM21 | 1410922 | 40 | 92.4 | 60/12 |
| BM22 | 1,020,178 | 28 | 77.1 | 215/25 |

*Samples sequenced by using Illumina HiSeq 2000.
